# HSC-derived exosomal miR-122-5p inhibits EMT and fibrosis of intrahepatic biliary epithelial cells to alleviate primary biliary cholangitis

**DOI:** 10.3389/fimmu.2025.1684064

**Published:** 2025-10-31

**Authors:** Yaqin Zhang, Ruofei Chen, Xueqing Yang, Long Qian, Bing Shen, Zongwen Shuai

**Affiliations:** ^1^ Department of Rheumatology, The Second Affiliated Hospital of Anhui Medical University, Hefei, China; ^2^ Department of Rheumatology, The First Affiliated Hospital of Anhui Medical University, Hefei, China; ^3^ State Key Laboratory of Chinese Medicine Quality Research, Macau University of Science and Technology, Macau, China

**Keywords:** primary biliary cholangitis, intrahepatic biliary epithelial cells, miRNA, exosomes, TNFRSF19/ASK1/p38 MAPK axis

## Abstract

**Introduction:**

Primary biliary cholangitis (PBC) is a chronic autoimmune-mediated cholestatic liver disease that can progress to cirrhosis and liver failure. Intrahepatic biliary epithelial cells (IBECs) are the primary targets of early injury in PBC. Our previous studies have shown that exosomes derived from hepatic stellate cells (HSCs) deliver miR-122-5p to regulate the expression of human IBEC inflammatory factors via the p38 MAPK signaling pathway. The purpose of this study was to investigate the therapeutic potential and molecular mechanism of HSC-derived exosomal miR-122-5p in PBC.

**Methods:**

The effects of exosomal miR-122-5p in inhibiting apoptosis, epithelial–mesenchymal transition (EMT), and fibrosis were evaluated in lipopolysaccharide (LPS)-induced human IBEC models, and its anti-inflammatory and anti-fibrotic effects were measured in dnTGF-βRII mouse models. A variety of analytical procedures, such as flow cytometry, Cell Counting Kit-8 (CCK-8), RT-qPCR, ELISA, co-culture, Western blotting, immunofluorescence, gene transfection, immunohistochemistry, and several staining methods (H&E and Masson), were used to evaluate the effectiveness and mechanisms of these methods.

**Results:**

The results from clinical data showed that exosomal miR-122-5p was correlated with liver function, and when combined with gp210 and sp100 antibodies, it could improve the sensitivity of PBC diagnosis. The results from *in vitro* experiments showed that exosomal miR-122-5p promoted the proliferation and inhibited the apoptosis, EMT, and fibrosis indicators of IBECs via the p38 MAPK signaling pathway. Dual luciferase reporter assay indicated that tumor necrosis factor receptor superfamily (TNFRSF) 19 is a specific target of miR-122-5p and reduces ASK1 levels. The co-immunoprecipitation (Co-IP) experiment further indicates the interaction between TNFRSF19 and ASK1. *In vivo* results indicated that the degrees of inflammatory infiltration and fibrosis in liver tissues of both PBC patients and model mice were more severe than those of normal controls and were then alleviated with exosomal miR-122-5p treatment.

**Conclusion:**

In conclusion, exosomal miR-122-5p alleviates liver pathology in PBC by targeting the TNFRSF19/ASK1/p38 MAPK axis, highlighting its potential as both a diagnostic biomarker and a therapeutic target for PBC.

## 1 Introduction

Primary biliary cholangitis (PBC) is a chronic autoimmune-mediated cholestatic liver disease that can progress to cirrhosis and liver failure, but the exact etiology is unknown (1). At present, serum AMA-M2 is a specific marker for the diagnosis of PBC. In addition, there are clinical immunological indicators such as gp210 and sp100 antibodies. Serum AMA-M2 was negative in some patients, which was mainly diagnosed by liver biopsy (2). Ursodeoxycholic acid (UDCA) is a U.S. Food and Drug Administration (FDA)-approved first-line treatment (3). However, approximately 40% of patients have an incomplete therapeutic response and may progress to liver transplantation and even die (4). Therefore, the identification of novel and promising biomarkers and therapeutic targets is crucial for the diagnosis and treatment of PBC.

Intrahepatic biliary epithelial cells (IBECs) are epithelial cells arranged on the surface of the intrahepatic bile duct lumen. They form a complex network system—the bile duct tree—which is responsible for collecting, modifying, and transporting bile secreted by liver cells. In addition, intrahepatic bile duct epithelial cells also express specific pattern recognition receptors, which are part of the liver’s innate immune system and can recruit and activate immune cells. In PBC, IBECs are the main targets of autoimmune attack. After being attacked, the IBECs will secrete a large amount of pro-inflammatory factors and chemokines, continuously driving the process of inflammation and fibrosis. Persistent inflammation eventually leads to the apoptosis and necrosis of the IBECs, and the interlobular bile ducts are destroyed and disappear. This hinders the normal flow of bile, leading to cholestasis. The accumulated bile acids are toxic, further damaging liver cells and IBECs, thus forming a vicious cycle (5). Meanwhile, IBECs activate and promote the transformation of hepatic stellate cells (HSCs) into myofibroblasts, indirectly driving the occurrence of liver fibrosis. Some studies have proposed that IBECs may promote liver fibrosis cell population through epithelial–mesenchymal transition (EMT) (6–8), and preventing the development of IBEC EMT could control or even reverse liver fibrosis (9). HSCs are a type of interstitial cell located in the Disse space of the hepatic sinusoid. At rest, HSCs synthesize a small amount of normal extracellular matrix (ECM) and express matrix metalloproteinases (MMPs) and their inhibitors [tissue inhibitors of metalloproteinases (TIMPs)] to maintain the integrity of the basement membrane and normal ECM balance. In PBC, HSCs are activated to synthesize and secrete a large amount of fibrous collagen, such as type I and type III collagen, while inhibiting the expression of MMPs and increasing the expression of TIMPs, leading to the excessive deposition of ECM, ultimately resulting in liver fibrosis (10). MicroRNA (miRNA) is a class of non-coding single-stranded RNA molecules approximately 19–24 nucleotides in length. By binding to target mRNA, it regulates gene expression and influences various physiological and pathological processes. Previous literature has reported that certain specific miRNAs (such as miR-506 and miR-425) are involved in the injury, inflammation, and fibrosis processes of IBECs. Therefore, miRNA has become a highly promising disease biomarker and a new therapeutic target (3, 11). In a previous study, we used lipopolysaccharide (LPS) treatment of human IBECs to mimic the state of IBECs in PBC patients, and we found that miR-122-5p level was decreased in LPS-induced IBECs (12). Studies have reported that loss of the miR-122 gene will lead to spontaneous liver fibrosis in mice (13, 14). A study established a mouse model of liver cirrhosis by intraperitoneal injection of carbon tetrachloride (CCl_4_) and miR-122 agomir (15). The results showed that after 8 weeks of CCl_4_ induction, the expression of miR-122 in the liver decreased, and the expression of α-SMA and collagen I in the liver tissue increased.

Our previous study (12) found the differential expression of miR-122-5p in serum exosomes of PBC patients and healthy controls by high-throughput sequencing and further confirmed that HSC-derived exosomal miR-122-5p can regulate the expression of inflammatory factors in IBECs through the p38 MAPK signaling pathway. In this study, we further clarified that exosomal miR-122-5p targets tumor necrosis factor receptor superfamily (TNFRSF) 19, regulates the p38 MAPK signaling pathway through ASK1, and inhibits the apoptosis, EMT, and fibrosis of IBECs. Additionally, using dnTGF-βRII mice (a PBC model), we investigated the effects of exosome-mediated overexpression of miR-122-5p on liver histopathological injury in mice, aiming to provide new insights and a scientific basis for interventional strategies in PBC.

## 2 Materials and methods

### 2.1 Clinical sample

Clinical sample collection, peripheral blood detection, and exosome extraction were the same as before (12). All patients with PBC met the diagnostic criteria of the 2018 American Association for the Study of Liver Diseases (16) and had not received any related drug treatment. The exclusion criteria were as follows: 1) severe heart, liver, and renal insufficiency; 2) acute diseases, such as fever, various infections, and acute myocardial infarction; 3) hepatitis A, B, C, E, etc.; 4) associated with other autoimmune diseases, such as systemic lupus erythematosus (SLE), rheumatoid arthritis (RA), systemic sclerosis (SS), vasculitis, and ankylosing spondylitis (AS); 5) various malignant tumors; and 6) fatty liver, gallstones, or underwent cholecystectomy. Ten pairs of liver tissues from PBC patients and the control group (adjacent tissues of hepatocellular carcinoma) were collected from the Department of Pathology of the First Affiliated Hospital of Anhui Medical University for routine hematoxylin and eosin (H&E), Masson staining, and immunohistochemical staining (17, 18). This study was approved by the Ethics Committee of the First Affiliated Hospital of Anhui Medical University, and all subjects signed the informed consent form (No. PJ2022-06-45).

### 2.2 Culture and co-culture of HSCs and IBECs

The culture of human HSCs and IBECs was the same as before (12). Mouse HSCs were purchased from Beina Chuanglian Biotechnology Co., Ltd., Beijing (China; Cat# C359737). Cultures were grown under standard conditions (37°C, 5% CO_2_) in Dulbecco's Modified Eagle’s Medium (DMEM) containing 10% fetal bovine serum and 1% penicillin and streptomycin solution. Cell passage was performed when the cells reached 70%–80% confluence. Cells at passages 3–6 were used for experiments. To investigate the effect of HSCs on IBECs, HSCs were seeded in the 0.4-μm polyester membrane Transwell inserts suitable for six-well plates, and the Transwell chamber containing HSCs was inserted into the six-well plate for co-culturing with IBECs for 24h.

### 2.3 Cell transfection

Overexpression was achieved by TNFRSF19 plasmid, ASK1 plasmid, and miR-122-5p mimics; knockdown was achieved by TNFRSF19 small interfering RNA (siRNA), ASK1 inhibitor, and miR-122-5p inhibitor. They were transfected using Lipofectamine 2000 (Invitrogen, USA, Carlsbad, California) according to the manufacturer’s protocol. If LPS was added, the medium was replaced with complete medium containing 100 ng/mL after 24h. The concentration of p38 inhibitor SB03580 was 500 nM, and the treatment time was 24h. The ASK1 inhibitor NQDI-1 was used at a concentration of 3 μM, and the treatment time was 24h. The plasmids and siRNA were designed by Sangon Biotech (Shanghai, China). The mimics and inhibitors were designed by GenePharma (Shanghai, China).

### 2.4 Flow cytometry

Cells were treated with pancreatic enzyme (Biyun Tian Biotech, Shanghai, China; Cat# C0201) and phosphate buffered saline (PBS) (Seville Biotech, Wuhan, China; Cat# SH30256.01); 400 μL propidium iodide (PI) dye solution was added, and the cells were stained at 4°C for 30–60 min away from light. According to the apoptosis kit instructions, cells were collected and centrifuged (1,500 rpm × 3min), and 5 μL allophycocyanin (APC) and 10 μL PI were added. Then, proliferation and apoptosis were tested using flow cytometry (NovoCyte). Flow cytometry images of apoptosis were analyzed using the NovoExpress software. Q1 represented necrotic cells, Q2 late apoptotic cells, Q3 normal cells, and Q4 early apoptotic cells. Apoptosis rate = Q2 + Q4. The cell cycle and apoptosis kit was purchased from Bebo Bio, Nangjing, China (BB-4104).

### 2.5 Cell viability assay

The activity of IBECs was detected using Cell Counting Kit-8 (CCK-8); 10 μL CCK-8 (Servicebio, Wuhan, China; Cat# G4103) was added to each well and cultured for 1h. Enzyme-linked immunosorbent assay (ELISA; RT6100) was used to measure the light absorption value of each hole at optical density (OD) 450 nm.

### 2.6 TUNEL staining

The coverslips were immersed in permeabilization solution and incubated at 37°C for 10min, followed by three 5-min washes with PBS. To each sample, 100 μL of buffer was applied and equilibrated in a humidified chamber at 37°C for 10–30 min. The buffer was removed using absorbent paper; 50 μL of TUNEL detection fluid (Cat# E-CK-A321; Elabscience Biotech, Wuhan, China) was added and incubated at 37°C for 60min. Diamidino-2'-PhenylIndole (DAPI) was used for nuclear counterstaining, and slides were observed under a microscope (Olympus, Naganuma, Hokkaido, Japan; CX43). The nuclei stained with DAPI appeared blue, while the positive expression appeared green.

### 2.7 RNA isolation and quantitative real‐time PCR

Total RNA from cells and tissues was extracted using TRIzol (Cat# 15596-026; Invitrogen, USA), and RNA concentrations were determined using a spectrophotometer (Thermo Fisher Scientific, Waltham, MA, USA). The RNA was reverse transcribed using cDNA Reverse Transcription Kits (Cat# 11141ES60, Yeasen Company, Shanghai, China). Relative gene expression levels were assessed with an ABI QuantStudio 6 Pro Real-Time PCR System using a SYBR mixture (Cat# 11202ES08; Yeasen Company, China). To quantify the relative level of miR-122-5p, a Hairpin-it™ miRNA RT-PCR Quantitation and U6 normalization kit (Cat# E22005; GenePharma, China) was used. The 2^−ΔΔCt^ method was applied to calculate the expression levels of mRNA and miRNA relative to the endogenous control genes, β-actin/18S ribosomal RNA and U6 small nuclear RNA, respectively. All primers were synthesized by Sangon Biotech (Shanghai, China) and are shown in **SI Appendix, Materials and Methods**-**Supplementary Table S1**.

### 2.8 Western blotting

Cells or tissues were homogenized in radioimmunoprecipitation assay buffer (Cat# P0013B; Beyotime Biotech, Shanghai, China), and protein concentrations were determined using bicinchoninic acid protein assay (Cat# P0010; Beyotime Biotech, China). Equal amounts of protein were loaded, separated by 10% sodium dodecyl sulfate–polyacrylamide gel electrophoresis, and transferred to polyvinylidene difluoride membranes (Millipore, Burlington, Massachusetts, USA). The membranes were blocked in 5% skim milk at room temperature for 1h and incubated overnight at 4 °C with primary antibodies. The membranes were then extensively washed in Tris-buffered saline with 0.1% Tween^®^ 20 detergent (TBST) and incubated with secondary antibodies for 2h at room temperature. After being washed three times with TBST, protein bands were detected using an enhanced chemiluminescence detection system (Bio-Rad Laboratories, Inc., Hercules, California). Luminescence intensity was analyzed using ImageJ. The antibodies used are shown in **SI Appendix**-**Supplementary Table S2**.

### 2.9 Co-immunoprecipitation (CO-IP)

#### 2.9.1 Preparation of cell lysates

IBECs were washed twice with ice-cold PBS at 48h after transfection. The cells were then lysed on ice for 30min using RadioImmunoPrecipitation Assay (RIPA) lysis buffer. The lysates were centrifuged at 14,000 × *g* for 5min at 4 °C to collect the supernatant. Protein concentrations were determined using a Bicinchoninic Acid (BCA) protein assay kit, and all samples were adjusted to the same concentration.

#### 2.9.2 Immunoprecipitation

A total of 500 μg of protein lysate was incubated with 2 μg of TNFRSF19/ASK1 with gentle rotation overnight at 4°C. The following day, 20 μL of pre-washed Protein A/G agarose beads was added, and the incubation continued for an additional 4h at 4°C. The beads were pelleted by centrifugation and washed four times with ice-cold lysis buffer to remove non-specifically bound proteins.

#### 2.9.3 Elution and Western blotting analysis

The immunocomplexes were eluted from the beads by boiling in 100 μL SDS–PAGE loading buffer for 5min. The eluted proteins were separated using sodium dodecyl sulfate–polyacrylamide gel electrophoresis (SDS–PAGE) and transferred to a polyvinylidene difluoride (PVDF) membrane. After blocking with 5% non-fat milk, the membrane was incubated with the indicated primary antibodies (anti-TNFRSF19/ASK1 antibody, 1:1,000; Affinity, Nangjing, China/Proteintech, Rosemon, Illinois, USA, Cat# DF13610; Cat# 28201-1-AP) overnight at 4°C, followed by incubation with appropriate horseradish peroxidase (HRP)-conjugated secondary antibodies (1:10,000; Zs-BIO, Beijing; Cat# ZB-2301 and Cat# ZB-2305) for 2h at room temperature. The protein signals were visualized using an ElectroChemiLuminescence (ECL) chemiluminescence kit and an imaging system.

### 2.10 Dual luciferase reporter assay

Wild-type (WT) human TNFRSF19 3′-UTR and mutant (mut) TNFRSF19 3′-UTR containing miR-122-5p binding sites were generated using the Site-Directed Mutagenesis Kit (General Biotech, Chuzhou, China). The mutant TNFRSF19 3′-UTR did not bind to miR-122-5p, while the WT did. The HEK-293 cell line has high transfection efficiency and stable expression of proteins. Therefore, the HEK-293 cell line was used to verify whether TNFRSF19 was truly targeted by miR-122-5p. HEK-293 cells were seeded in 12-well format dishes at 2 × 10^5^/well and co-transfected with luciferase reporter vectors TNFRSF19-wt, TNFRSF19-mut, and miR-122-5p mimics and mimics NC. Relative luciferase activities were measured at 48h after transfection using a dual luciferase reporter assay system (Beyotime Biotech, Shanghai, China) according to the manufacturer’s protocol.

### 2.11 Immunofluorescence

IBECs were cultured in 12-well plates containing glass slides and treated for 24h. Cells were then fixed with 4% paraformaldehyde at room temperature for 30min, permeabilized with 0.1% Triton X-100 (Cat# P0096; Beyotime Biotech, Shanghai, China) at 4°C for 20min, and washed with PBS. Non-specific fluorescence was blocked using immunofluorescence-specific blocking solution (Cat# P0260; Beyotime Biotech, Shanghai, China). Primary antibodies for TNFRSF19 (Cat# 82t9786; Affinity, China) and ASK1 (Cat# 29r3489; Affinity, China) were used for overnight staining at 4°C. After washing with PBS, cells were co-incubated with fluorophore-conjugated secondary antibodies (Abcam, Cambridge, UK). Finally, cells were mounted with DAPI-containing mounting medium (Cat# P0131; Beyotime Biotech, Shanghai, China), and images were acquired using a fluorescence microscope (Cat# DMI8; Leica, Wetzlar, Germany). DAPI ultraviolet excitation wavelength is 330–380 nm, and emission wavelength is 420 nm, emitting blue light. CY3 has an excitation wavelength of 510–560 and an emission wavelength of 590 nm, emitting red light. The fluorescein isothiocyanate (FITC) fluorophore has a peak excitation wavelength of 495 nm and a peak emission wavelength of 519 nm, producing green fluorescence.

### 2.12 Exosome extraction of mouse HSCs

Mouse HSCs were seeded in blank plates and transfected with mmu-miR-122-5p. After 6h, the medium was replaced with complete medium, and the cells were cultured for 48h. The medium was removed, and 2 mL of immune cell serum-free medium was added to each well and cultured for 48h. A total of 300 mL of cell supernatant was collected. Then, exosomes were isolated from cell supernatant using an exosome kit (Cat# 084001; Beibei Biotech, Zhengzhou, China), with one aliquot of exosomal precipitate obtained per 50 mL of supernatant. Exosomes were labeled with PKH26 red fluorescent cell membrane staining kit (Cat# D0030; Solarbio, Beijing, China). The extracted exosome precipitate was resuspended in 100 μL of dilution C, and 100 μL of 2× staining solution containing 4 × 10^−6^ M PKH26 was added.

### 2.13 Exosome intake experiment

The exosomes derived from HSCs were labeled with red PKH26 fluorescent membrane dye (Solarbio, China). IBEC nuclei were labeled using blue fluorescent DAPI. The labeled exosomes were added to the culture medium of IBECs at a concentration of 90 μg/mL. After marking different times, they were washed twice with PBS and photographed under different fields of view (×200) using an inverted microscope (Olympus).

### 2.14 Breeding and identification of mice

The PBC model mice used in this study were dnTGF-βRII mice, which were a gift from Professor Lian Zhexiong, Institute of Life Sciences, South China University of Technology. DnTGF-βRII mice are PBC models constructed by overexpressing the dominant inactive form of TGF-β type II receptor (TGF-βRII) under the control of the promoter. It can spontaneously produce AMA, 100% AMA positive, extensive CD4^+^ and CD8^+^ lymphocyte infiltration in the portal vein, increased serum IFN-γ and TNF-α levels, and interlobular bile duct damage, which can satisfactorily mimic the clinical phenotype of the disease. It is currently recognized as an animal model of PBC. Mice were bred in the Specific Pathogen-Free (SPF) animal house of Jiangsu Jichi Pharmaceutical Kang Biotechnology Co., Ltd. C57BL/6J female and dnTGF-βRII male mice (over 8 weeks old) were bred according to the ratio of one male to two females and separated after 1 week; 2–3-mm tails were cut from the born mice at 3 weeks old, and the deoxyribonucleic acid (DNA) was extracted using alkaline cleavage. The mouse genes were identified using PCR and electrophoresis separation, and the positive female mice were retained. The gene was identified as follows. Primer sequence dnTg-F: GCTGCACATCGTCCTGTG; PCR amplification conditions, 94°C for 3 min; WT (bp), 324; mutant (bp), 100. Primer sequence dnTg-R: ACTTGACTGCACCGTTGTTG; PCR amplification conditions, 94°C for 30 s, 60°C for 1min, 72°C for 1min, and 30 cycles. Primer sequence dnTg-wtF: CTAGGCCACAGAATTGAAAGATCT; PCR amplification conditions, 72°C for 2 min. Primer sequences dnTg-wtR: GTAGGTGGAAATTCTAGCATCATCC; PCR amplification was performed at 10°C. The genetic identification of some PBC model mice is shown in **SI Appendix**-**Supplementary Figure S13**. The animal experiment was approved by the Ethics Committee of Anhui Medical University (LLSC20221111).

### 2.15 Mouse tail vein injection and *in vivo* imaging

DnTGF-βRII female mice were raised to 12–14 weeks old (20–30 g, fed and watered freely 24h before the experiment). According to the results of the preliminary experiment (**SI Appendix**-**Supplementary Figure S14**), 50 μL of exosomes (extracted from 50 mL of mouse HSC supernatant loaded with miR-122-5p) was injected into the tail vein. After 48h, five mice were placed into the imaging dark box platform (Ivis Spectrum Andor Camera, IS2329N9250), and the appropriate excitation and emission filters were selected. The exosomes carried CY3 fluorescence, with an excitation wavelength of 550 nm and an emission wavelength of 570 nm.

### 2.16 Histopathology

All 10 dnTGF-βRII mice and five C57BL/6J mice were sacrificed, and peripheral blood and liver tissues were collected. Liver tissues were excised immediately upon sacrifice; one was fixed in 4% paraformaldehyde (PFA) solution for 2 days at room temperature, embedded in paraffin, and cut into 4-μm sections for Masson trichrome staining and H&E staining, according to standard protocols. The inflammation of the tissue was assessed according to H&E staining by the following two parameters: severity and frequency. The final scores for portal and lobular inflammation and bile duct damage were calculated as the sum of the indices for severity and frequency. Portal and lobular inflammation was evaluated as 0 = none, 1 = minimal, 2 = mild, 3 = moderate, and 4 = severe inflammation. Bile duct damage was evaluated as 0 = none, 1 = epithelial damage with cytoplasmic change, 2 = epithelial damage with nuclear change, 3 = chronic non-suppurative destructive cholangitis, and 4 = bile duct loss. Frequencies were scored as 1 = none, 2 = 1%–10%, 3 = 11%–20%, 4 = 20%–50%, and 5 = more than 50% frequency. Fibrosis of the tissue was evaluated according to Masson staining (collagen fibers are blue). The color deconvolution function in Fiji was used to distinguish and identify different colors. The separation parameters for Masson staining were set in Fiji, and the blue area was selected using the threshold function. The gray values were converted to OD values, and the parameters were indicated for measurement. All sections were evaluated by three pathologists who were blind to the design of the study (18, 19).

### 2.17 Immunohistochemistry

Liver tissues were fixed in 4% paraformaldehyde, followed by paraffin embedding and sectioning into 5-μm slices. The sections underwent deparaffinization and antigen retrieval. Primary antibodies for TNFRSF19, ASK1, and p-p38 were used to measure expression levels (**SI Appendix**-**Supplementary Table S2**). All animal procedures were approved by the Animal Care Committee of the First Affiliated Hospital of Anhui Medical University (LLSC20221111).

### 2.18 Statistical analysis

Normal distribution data were presented as mean ± standard deviation (SD). The measurement data were performed using Student’s *t*-test (two groups) and one-way ANOVA (more than two groups), followed by Bonferroni’s *post-hoc* tests. When n < 5, statistical analysis was performed using permutation tests. The count data were compared using the chi-square (χ^2^) test. Spearman’s correlation was used to analyze the correlation between exosomal miR-122-5p and liver function. The R software 4.3.0 pROC package and the glmnet package were used to construct diagnostic predictions for single and combined indicators and to compare area under the curve (AUC) values.

## 3 Result

### 3.1 Clinical value of exosomal miR-122-5p

**Figure 1:**
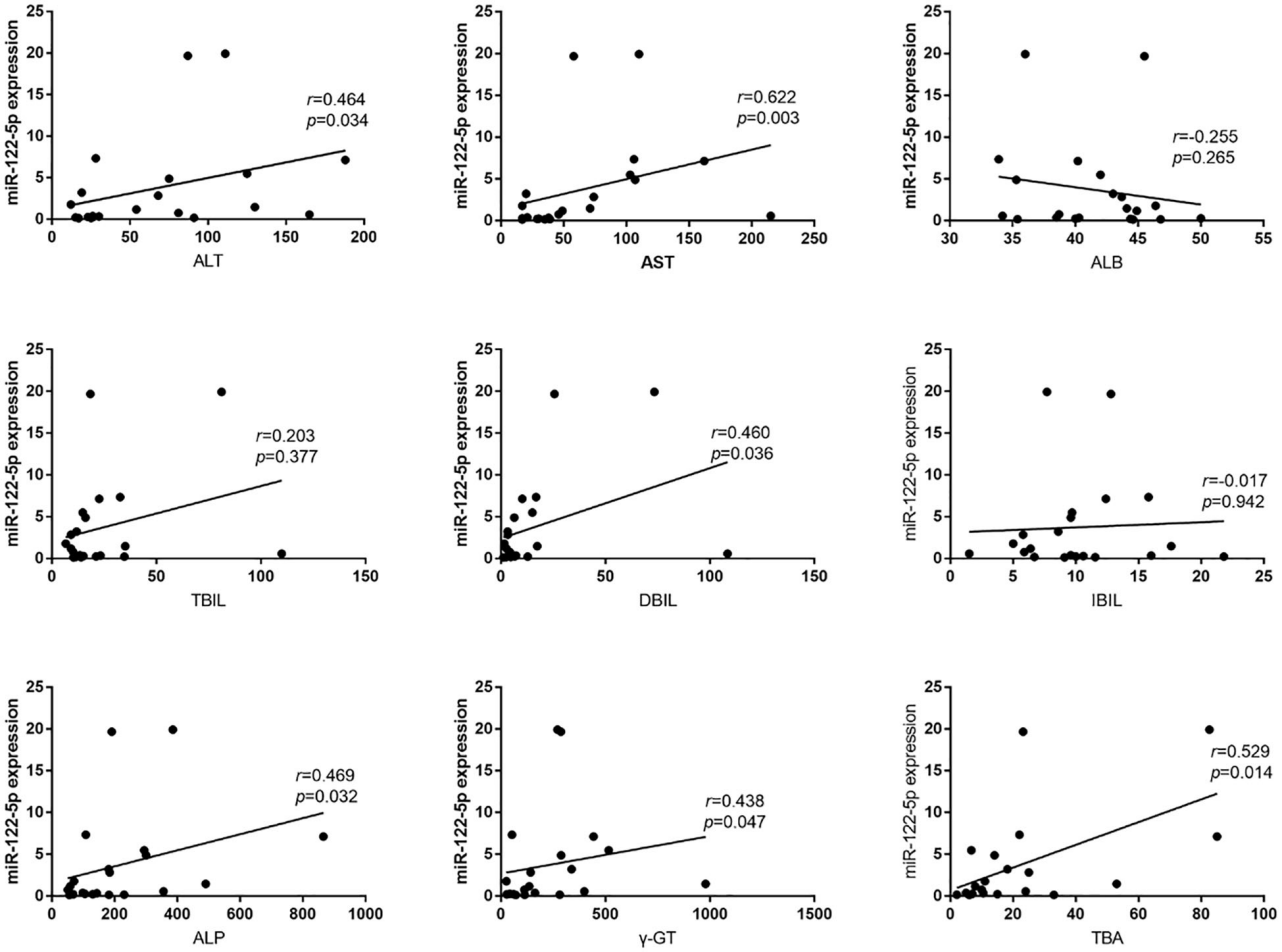
Relationship between exosomal miR-122-5p and clinical biochemical indices. ALT, alanine aminotransferase; AST, aspartate aminotransferase; ALB, albumin; TBIL, total bilirubin; IBIL, indirect bilirubin; DBIL, direct bilirubin; TBA, total bile acid; ALP, alkaline phosphatase; γ-GT, γ-glutamyl transpeptidase; *r*, correlation coefficient.

According to the inclusion and exclusion criteria, from January 2021 to January 2023, we collected a total of 21 serum samples from PBC patients and 22 controls. To further understand the relationship between exosomal miR-122-5p and liver injury and cholestasis in patients with PBC and its diagnostic value, Pearson’s correlation analysis was performed, which showed that the level of exosomal miR-122-5p was positively correlated with alanine aminotransferase (ALT), aspartate aminotransferase (AST), direct bilirubin (DBIL), γ-glutamyl transpeptidase (γ-GT), alkaline phosphatase (ALP), and total bile acid (TBA) (*p*<0.05), but had no correlation with albumin (ALB), total bilirubin (TBIL), and indirect bilirubin (IBIL) (**Figure 1**).

**Figure 2:**
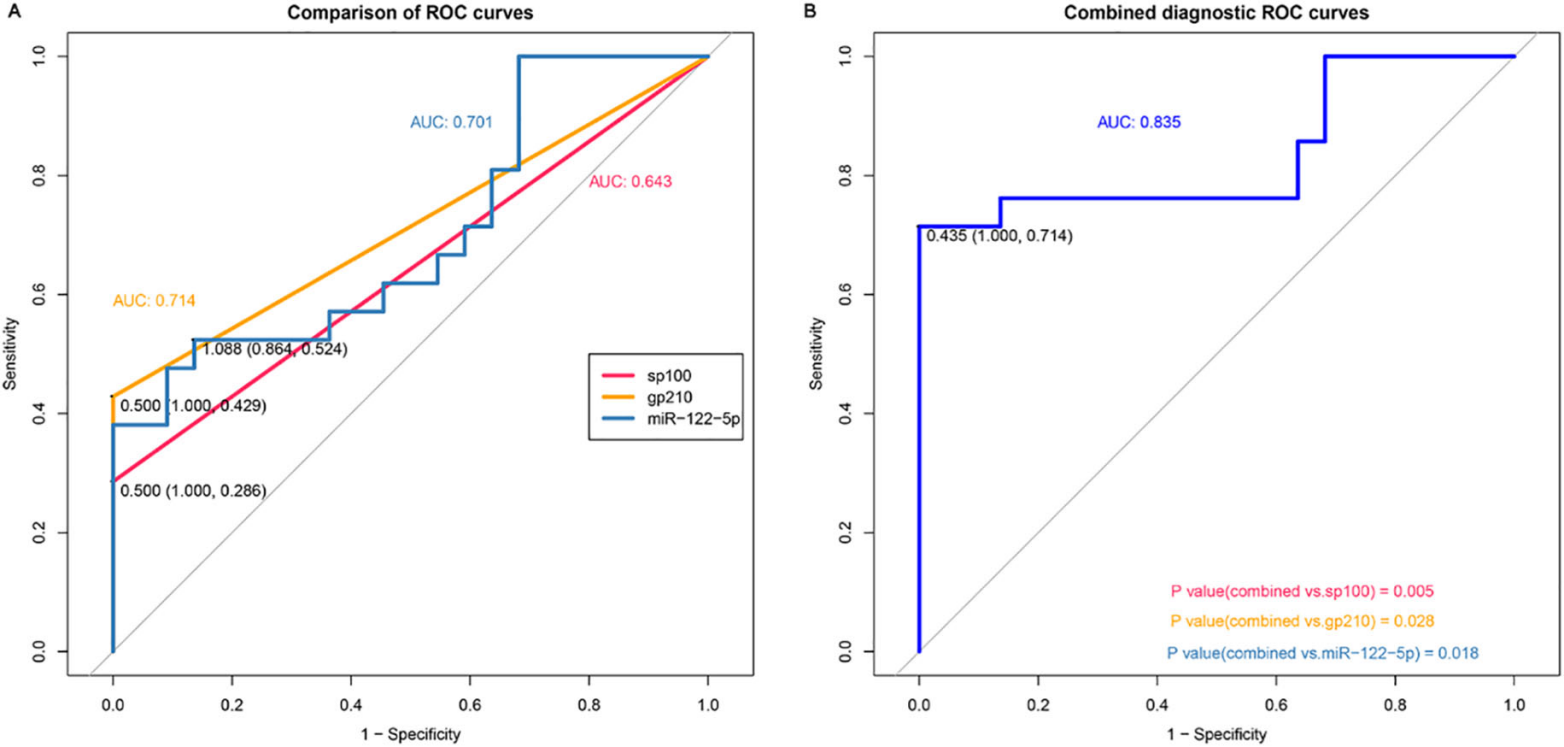
Diagnostic value of exosomal miR-122-5p for PBC. **(A)** Area under the curve of exosomal miR-122-5p, sp100, and gp210. **(B)** Area under the curve of exosomal miR-122-5p combined with sp100 and gp210. AUC, area under the curve. The cohort for ROC analysis consists of n = 21 PBC and n = 22 controls. PBC, primary biliary cholangitis.

The receiver operating characteristic (ROC) curves showed that the AUC of exosomal miR-122-5p was 0.701 (95% CI: 0.541–0.862, *p*=0.995), and the cut-off values (sensitivity, specificity) were 1.088 (0.524, 0.864). The AUC values of sp100 and gp210 antibodies were 0.643 (95% CI: 0.544–0.742, *p*=0.995) and 0.714 (95% CI: 0.606–0.823, *p*=0.994), respectively, and their cut-off values (sensitivity, specificity) were 0.500 (0.286, 1.000) and 0.500 (0.429, 1.000), respectively (**Figure 2A**). The AUC of exosomal miR-122-5p combined with sp100 and gp210 antibodies was 0.835 (95% CI: 0.73–0.968), and the cut-off values (sensitivity, specificity) were 0.435 (0.714, 1.000). The combined AUC was higher than that of any of the above single indicators, and the difference was statistically significant, which improved the sensitivity of diagnosing PBC (**Figure 2B**).

### 3.2 Exosomal miR-122-5p promoted the proliferation and inhibited the apoptosis, EMT, and fibrosis of IBECs

**Figure 3:**
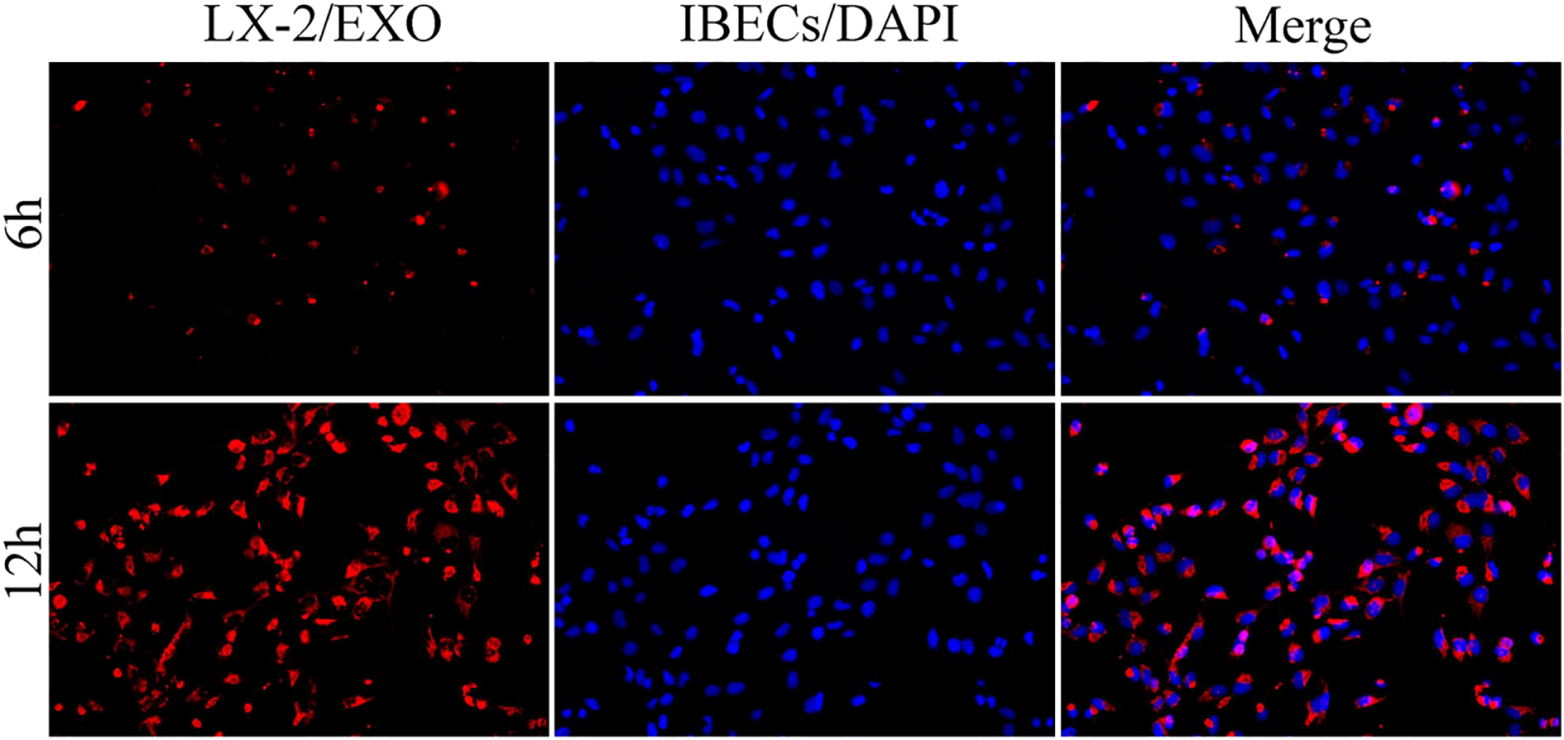
HSC exosomes were taken up by IBECs as observed under inverted fluorescence microscope (×200) at 6 and 12h. Red fluorescence is the exosomes labeled with PKH26 dye, blue fluorescence is the IBEC nucleus, and Merge is the combination picture. HSC, hepatic stellate cell; IBECs, intrahepatic biliary epithelial cells.

In previous studies, we have demonstrated that exosomes derived from HSCs deliver miR-122-5p to regulate the levels of IBEC inflammatory factors via the p38 MAPK signaling pathway. In this study, we co-cultured human HSCs (LX-2 cells) and IBECs using a Transwell chamber and labeled exosomes with PKH26. After 6 and 12h, we conducted an observation using an inverted fluorescence microscope. We found that exosomes derived from HSCs could be taken up by IBECs. Moreover, at 12h, the uptake of exosomes by IBECs increased significantly (**Figure 3**).

**Figure 4:**
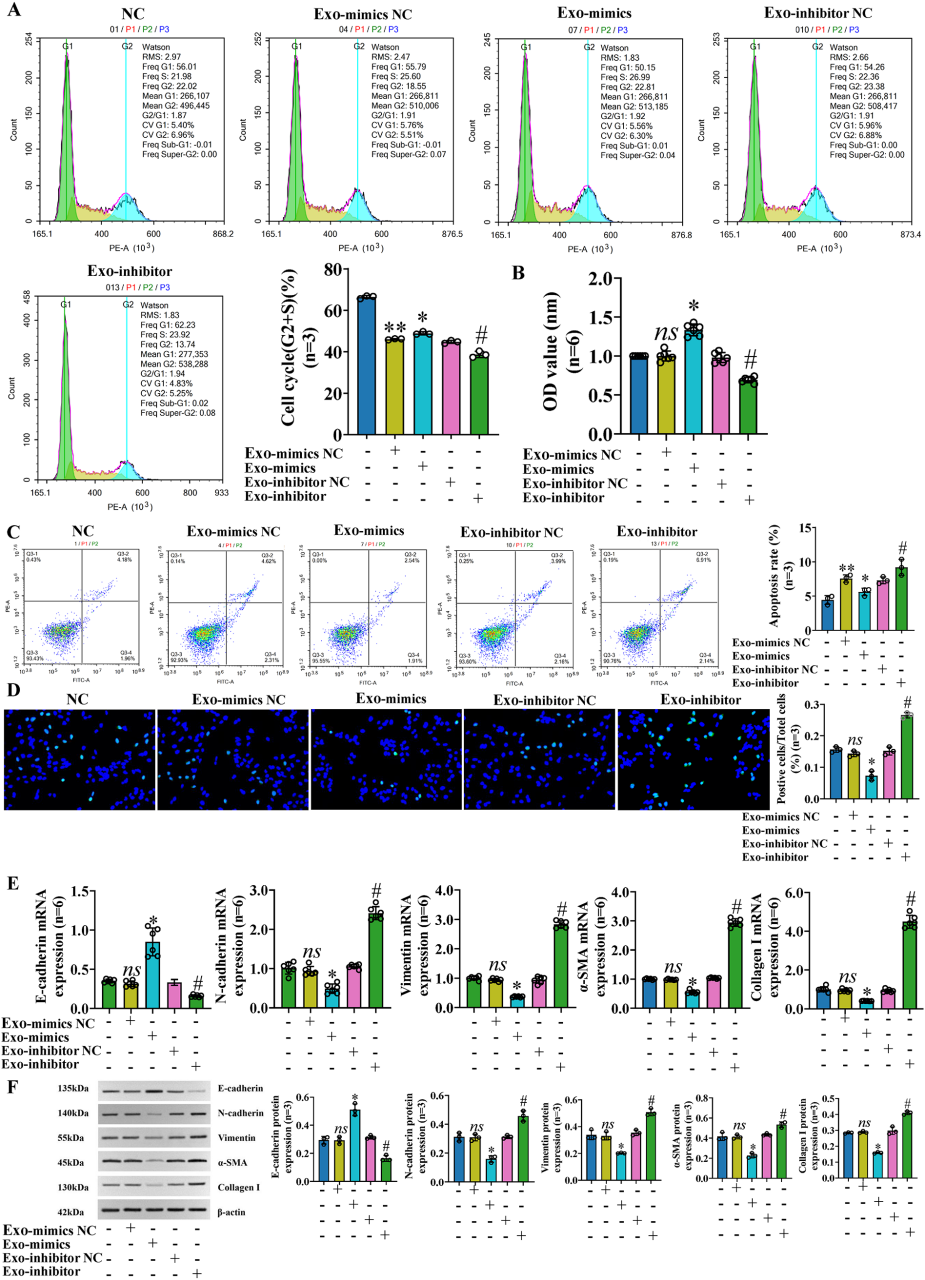
Exosomal miR-122-5p promoted the proliferation and inhibited the apoptosis, EMT, and fibrosis of IBECs. **(A)** Flow cytometry was used to detect the cycle of IBECs. **(B)** CCK-8 was used to detect the viability of IBECs. **(C, D)** Flow cytometry and TUNEL staining (×200) were used to detect the apoptosis of IBECs. **(E, F)** RT-qPCR and Western blotting were used to assess the mRNA and protein levels of EMT- and fibrosis-related markers in IBECs. *ns*, no significance; *p<0.05, compared to Exo-mimics NC group; ^#^ *p*<0.05, compared to Exo-inhibitor NC group; **p<0.01, compared to NC group. NC: untreated IBECs; Exo-mimics NC: exosomes transfected with miR-122-5p mimics NC were added to the IBEC culture medium; Exo-mimics: exosomes overexpressing miR-122-5p were added to the IBEC culture medium; Exo-inhibitor NC: exosomes transfected with miR-122-5p inhibitor NC were added to the IBEC culture medium; Exo-inhibitor: exosomes transfected with miR-122-5p inhibitor were added to the IBEC culture medium. α-SMA, α-smooth muscle actin. Statistical analysis was performed (n ≥ 5) using one-way ANOVA followed by Bonferroni’s *post-hoc* tests; when n < 5, it was performed using permutation tests. EMT, epithelial–mesenchymal transition; IBECs, intrahepatic biliary epithelial cells; CCK-8, Cell Counting Kit-8.

**Figure 5:**
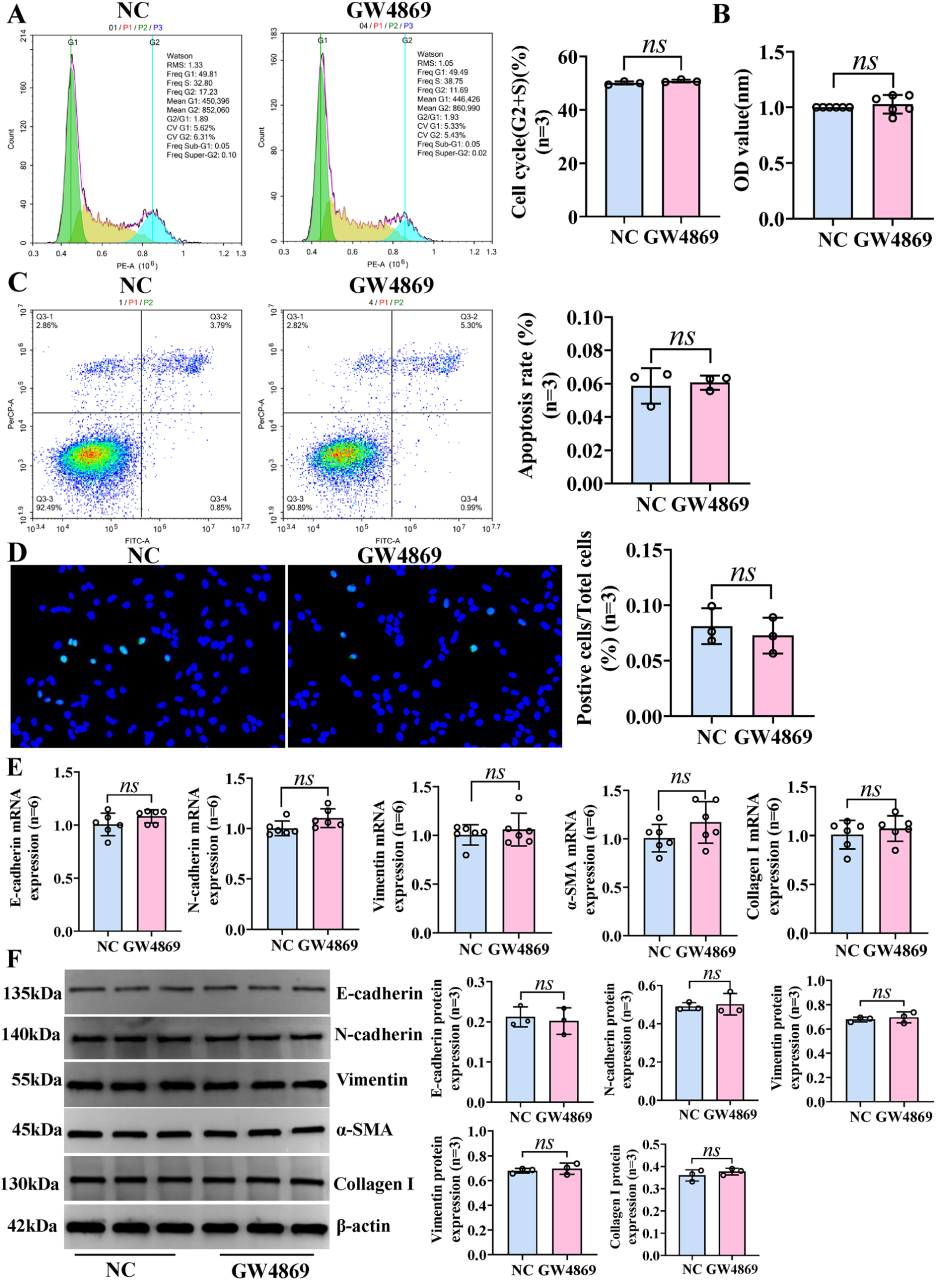
Exosomes have no effect on the phenotypic changes of IBECs. HSCs and IBECs were co-cultured using Transwell chambers. **(A)** Flow cytometry was used to detect the cycle of IBECs. **(B)** CCK-8 was used to detect the viability of IBECs. **(C, D)** Flow cytometry and TUNEL staining (×200) were used to detect the apoptosis of IBECs. **(E, F)** RT-qPCR and Western blotting were used to assess the mRNA and protein levels of EMT- and fibrosis-related markers in IBECs. *ns*, no significance. NC: untreated HSCs; GW4869: HSCs were treated with 10 μmol/L GW4869 for 24h. α-SMA, α-smooth muscle actin. Statistical analysis was performed (n ≥ 5) using one-way ANOVA followed by Bonferroni’s *post-hoc* tests; when n < 5, it was performed using permutation tests. HSCs, hepatic stellate cells; IBECs, intrahepatic biliary epithelial cells; CCK-8, Cell Counting Kit-8; EMT, epithelial–mesenchymal transition.

To further study the role and molecular mechanism of exosomal miR-122-5p in PBC, we transfected LX-2 with miR-122-5p, then extracted exosomes from the supernatants of LX-2 cells, and co-cultured them with IBECs to regulate the levels of miR-122-5p. The identification results of exosomes and methods were the same as before (12). The results showed that the exosomes with high expression of miR-122-5p promoted the proliferation and inhibited the apoptosis (**Figures 4A–D**) and EMT and fibrosis of IBECs (**Figures 4E, F**), as evidenced by increases in the cell cycle (G2+S), cell viability, and E-cadherin and decreases in the apoptosis rate, N-cadherin, vimentin, α-SMA, and collagen I of the Exo-mimics group, and the converse applies (**SI Appendix**, **Supplementary Figures S1**, **S2**). To further distinguish whether it was exosomes or exosome-mediated miR-122-5p that affected IBECs, we co-cultured the two again and divided them into two groups based on whether exosome blockers (GW4869, Cat# H-19363; MCE, Monmouth Junction, NJ, USA) were added. The results showed that there were no statistically significant differences in the proliferation, apoptosis (**Figures 5A–D**), EMT, and fibrosis indicators (**Figures 5E, F**) of IBECs between the two groups (**SI Appendix**, **Supplementary Figure S3**). It indicates that exosomes do not have an impact on the phenotypic changes of IBECs, and it is mainly the exosome-mediated miR-122-5p that plays a role.

### 3.3 The effect on IBECs of LPS was reversed by exosomal miR-122-5p

**Figure 6:**
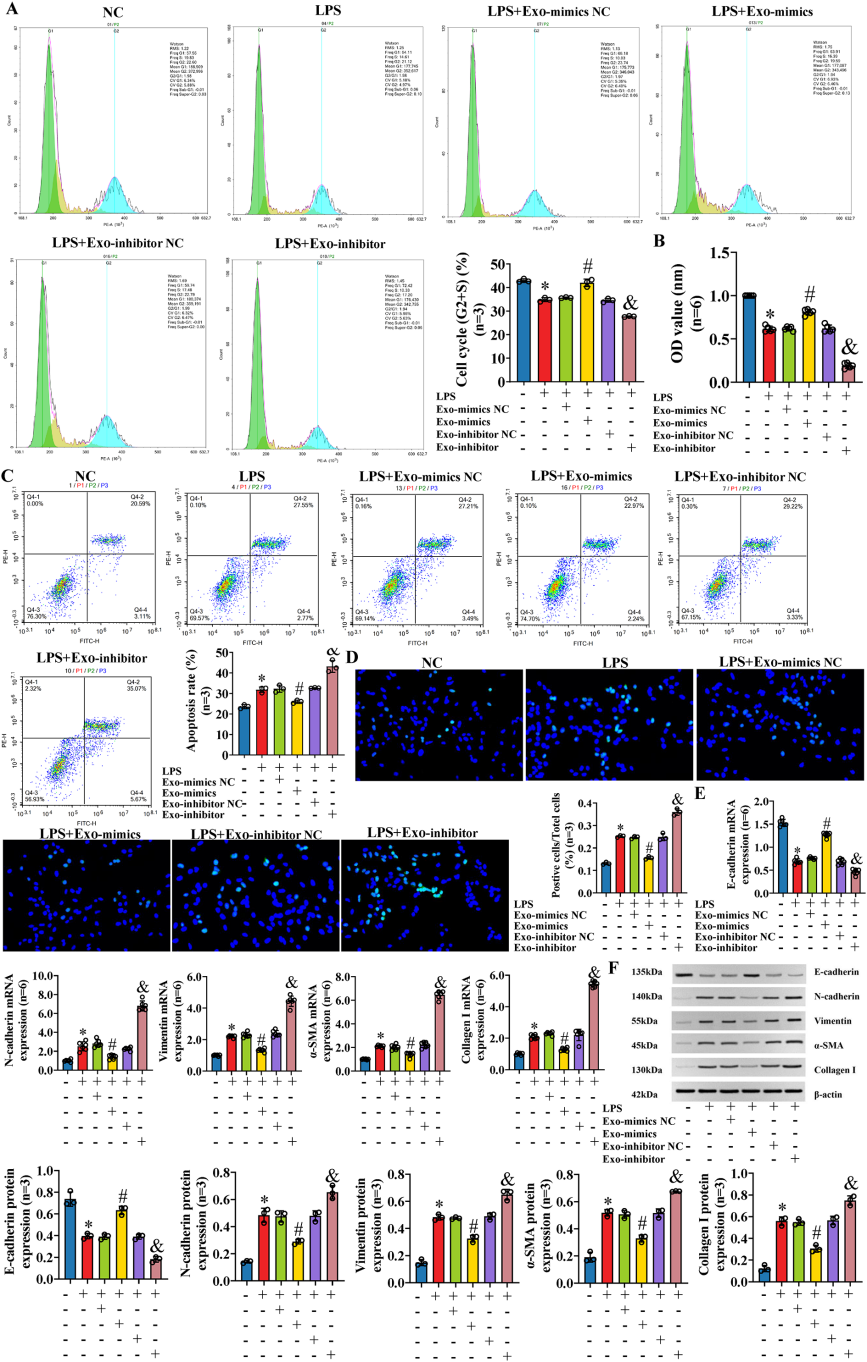
The effect on IBECs of LPS was reversed by exosomal miR-122-5p. **(A)** Flow cytometry was used to detect the cycle of IBECs. **(B)** CCK-8 was used to detect the viability of IBECs. **(C, D)** Flow cytometry and TUNEL staining (×200) were used to detect the apoptosis of IBECs. **(E, F)** RT-qPCR and Western blotting were used to assess the mRNA and protein levels of EMT- and fibrosis-related markers in IBECs. *p<0.05, compared to NC group; ^#^ *p*<0.05, compared to LPS+Exo-mimics NC group; ^&^ *p*<0.05, compared to LPS+Exo-inhibitor NC group. NC: untreated IBECs; LPS: 0.1 μg/mL LPS treated for 24h; Exo-mimics NC: exosomes transfected with miR-122-5p mimics NC were added to the IBEC culture medium; Exo-mimics: exosomes overexpressing miR-122-5p were added to the IBEC culture medium; Exo-inhibitor NC: exosomes transfected with miR-122-5p inhibitor NC were added to the IBEC culture medium; Exo-inhibitor: exosomes transfected with miR-122-5p inhibitor were added to the IBEC culture medium. α-SMA, α-smooth muscle actin. Statistical analysis was performed (n ≥ 5) using one-way ANOVA followed by Bonferroni’s *post-hoc* tests; when n < 5, it was performed using permutation tests. IBECs, intrahepatic biliary epithelial cells; LPS, lipopolysaccharide; CCK-8, Cell Counting Kit-8; EMT, epithelial–mesenchymal transition.

To further explore the effect of exosomal miR-122-5p on IBECs based on the inflammatory injury, exosomes derived from human LX-2 cells (transfected with miR-122-5p NC, miR-122-5p mimics, and miR-122-5p inhibitor) were co-cultured with LPS-induced IBECs. The results showed that LPS decreased the IBEC cycle (G2+S) ratio, cell viability, and E-cadherin and increased the apoptosis rate, N-cadherin, vimentin, α-SMA, and collagen I compared with those in the NC group. That is, LPS inhibited the proliferation and promoted the apoptosis (**Figures 6A–D**), EMT, and fibrosis of IBECs (**Figures 6E, F**). This effect was reversed by exosomes overexpressing miR-122-5p, evidenced by increases in the cell cycle (G2+S), cell viability, and E-cadherin and decreases in the apoptosis rate, N-cadherin, vimentin, α-SMA, and collagen I of the LPS+Exo-mimics group, compared to the LPS+Exo-mimics NC group. Conversely, the low expression of miR-122-5p in exosomes has a synergistic effect with LPS, evidenced by decreases in the cell cycle (G2+S), cell viability, and E-cadherin and increases in the apoptosis rate, N-cadherin, vimentin, α-SMA, and collagen I of the LPS+Exo-inhibitor group, compared to the LPS+Exo-inhibitor NC group (**SI Appendix**, **Supplementary Figures S4**, **S5**).

### 3.4 Exosomal miR-122-5p affects the proliferation, apoptosis, EMT, and fibrosis of IBECs via the p38 MAPK signaling pathway

**Figure 7:**
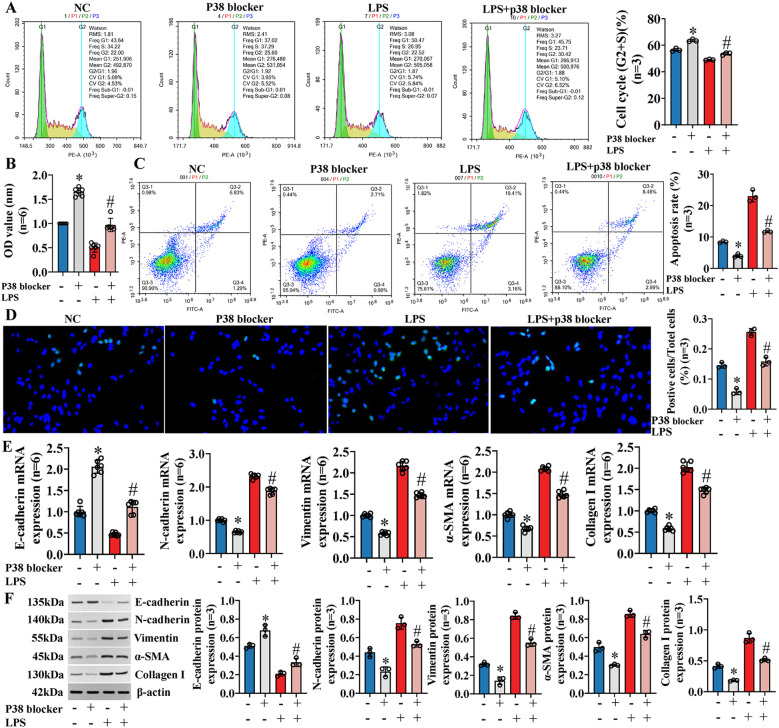
Exosomal miR-122-5p affects the proliferation, apoptosis, EMT, and fibrosis of IBECs via p38 MAPK signaling pathway. **(A)** Flow cytometry was used to detect the cycle of IBECs. **(B)** CCK-8 was used to detect the viability of IBECs. **(C, D)** Flow cytometry and TUNEL staining (×200) were used to detect the apoptosis of IBECs. **(E, F)** RT-qPCR and Western blotting were used to assess the mRNA and protein levels of EMT- and fibrosis-related markers in IBECs. *p<0.05, compared to NC group; ^#^ *p*<0.05, compared to LPS group. NC: untreated IBECs; LPS: 0.1 μg/mL LPS treated for 24h; p38 blocker: p38 MAPK pathway blocker SB03580 treated for 24h. α-SMA, α-smooth muscle. Statistical analysis was performed (n ≥ 5) using one-way ANOVA followed by Bonferroni’s *post-hoc* tests; when n < 5, it was performed using permutation tests. EMT, epithelial–mesenchymal transition; IBECs, intrahepatic biliary epithelial cells; CCK-8, Cell Counting Kit-8.

Previous studies (11) have shown that exosomal miR-122-5p can affect the expression of inflammatory factors in IBECs via the p38 MAPK signaling pathway. Similarly, we blocked the p38 MAPK pathway through SB03580, and the results showed that the IBEC cycle (G2+S) ratio, cell viability, and E-cadherin were increased and the apoptosis rate, N-cadherin, vimentin, α-SMA, and collagen I were decreased compared to those in the NC group (**Figures 7A–D**). Furthermore, the p38 blocker can partially reverse the effect of LPS on IBECs, which was basically consistent with the results of exosomes overexpressing miR-122-5p. That is, the IBEC cycle (G2+S) ratio, cell viability, and E-cadherin were increased and the apoptosis rate, N-cadherin, vimentin, α-SMA, and collagen I (**Figures 7E, F**) were decreased in the LPS+p38 blocker group, compared to the LPS group (**SI Appendix**, **Supplementary Figures S6**, **S7**).

**Figure 8:**
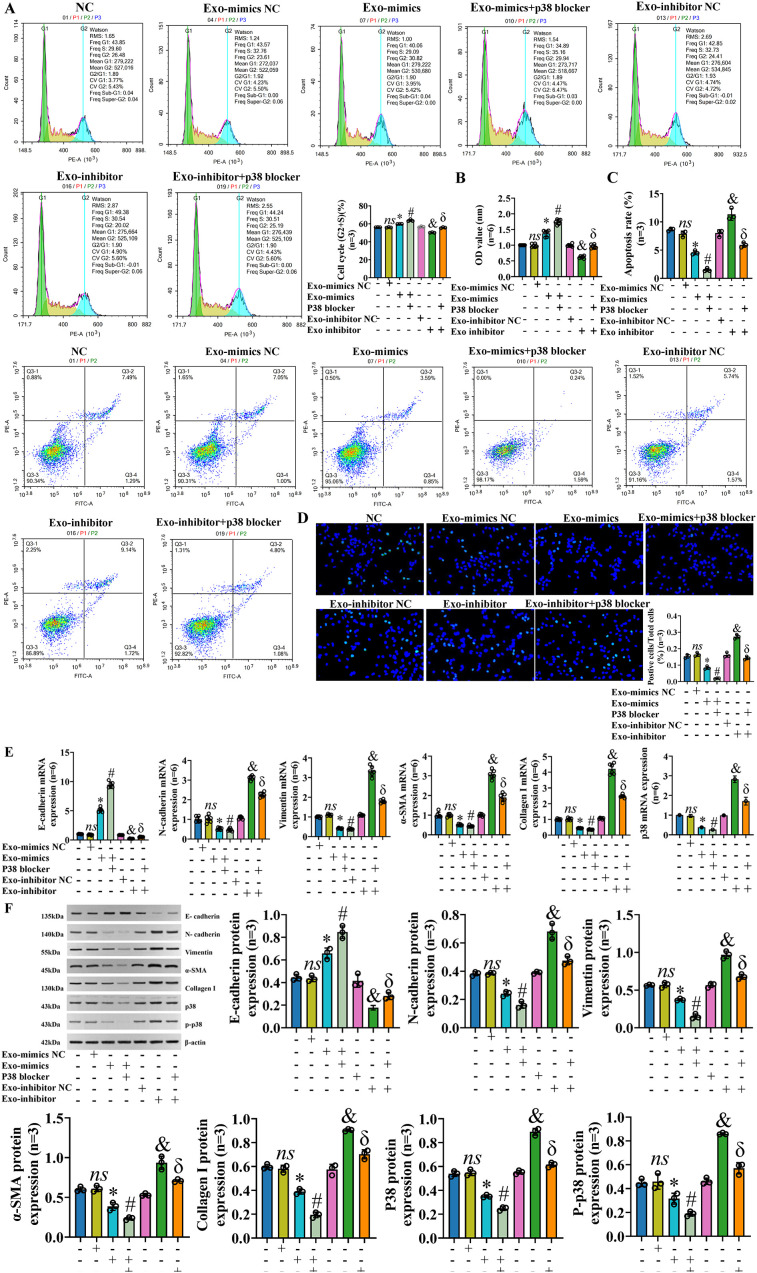
Exosomal miR-122-5p affects the proliferation, apoptosis, EMT, and fibrosis of IBECs via p38 MAPK pathway. *Notes*: **(A)** Flow cytometry was used to detect the cycle of IBECs. **(B)** CCK-8 was used to detect the viability of IBECs. **(C, D)** Flow cytometry and TUNEL staining (×200) were used to detect the apoptosis of IBECs. **(E, F)** RT-qPCR and Western blotting were used to assess the mRNA and protein levels of EMT- and fibrosis-related markers in IBECs. *ns*, no significance; *p<0.05, compared to Exo-mimics NC group; ^#^ *p*<0.05, compared to Exo-mimics group; ^&^ *p*<0.05, compared to Exo-inhibitor NC group; ^δ^ *p*<0.05, compared to Exo-inhibitor group. NC: untreated IBECs; Exo-mimics NC: exosomes transfected with miR-122-5p mimics NC were added to the IBEC culture medium; Exo-mimics: exosomes overexpressing miR-122-5p were added to the IBEC culture medium; Exo-inhibitor NC: exosomes transfected with miR-122-5p inhibitor NC were added to the IBEC culture medium; Exo-inhibitor: exosomes transfected with miR-122-5p inhibitor were added to the IBEC culture medium; p38 blocker: p38 MAPK pathway blocker SB03580 treated for 24h. α-SMA, α-smooth muscle actin. Statistical analysis was performed (n ≥ 5) using one-way ANOVA followed by Bonferroni’s *post-hoc* tests; when n < 5, it was performed using permutation tests. EMT, epithelial–mesenchymal transition; IBECs, intrahepatic biliary epithelial cells; CCK-8, Cell Counting Kit-8.

To further verify whether exosomal miR-122-5p really affects IBECs via the p38 MAPK signaling pathway, IBECs were co-transfected with exosomal miR-122-5p and p38 blockers. After the p38 MAPK signaling pathway was blocked, it played a synergistic role with exosomes that overexpressed miR-122-5p, but had a reverse effect with exosomes that had low expression of miR-122-5p, as evidenced by increases in the IBEC cycle (G2+S) ratio, cell viability, and E-cadherin expression and decreases in the apoptosis rate (**Figures 8A–D**), N-cadherin, vimentin, α-SMA, and collagen I in the Exo-mimics+p38 blocker group and Exo-inhibitor+p38 blocker group, compared to the Exo-mimics group and Exo-inhibitor group (**Figures 8E, F**), respectively (**SI Appendix**, **Supplementary Figures S8**, **S9**). All the above results indicated that exosomal miR-122-5p really affected the proliferation, apoptosis, EMT, and fibrosis of IBECs via the p38 MAPK signaling pathway.

### 3.5 Effects of exosomal miR-122-5p and p38 MAPK blocker in the IBEC inflammatory model

**Figure 9:**
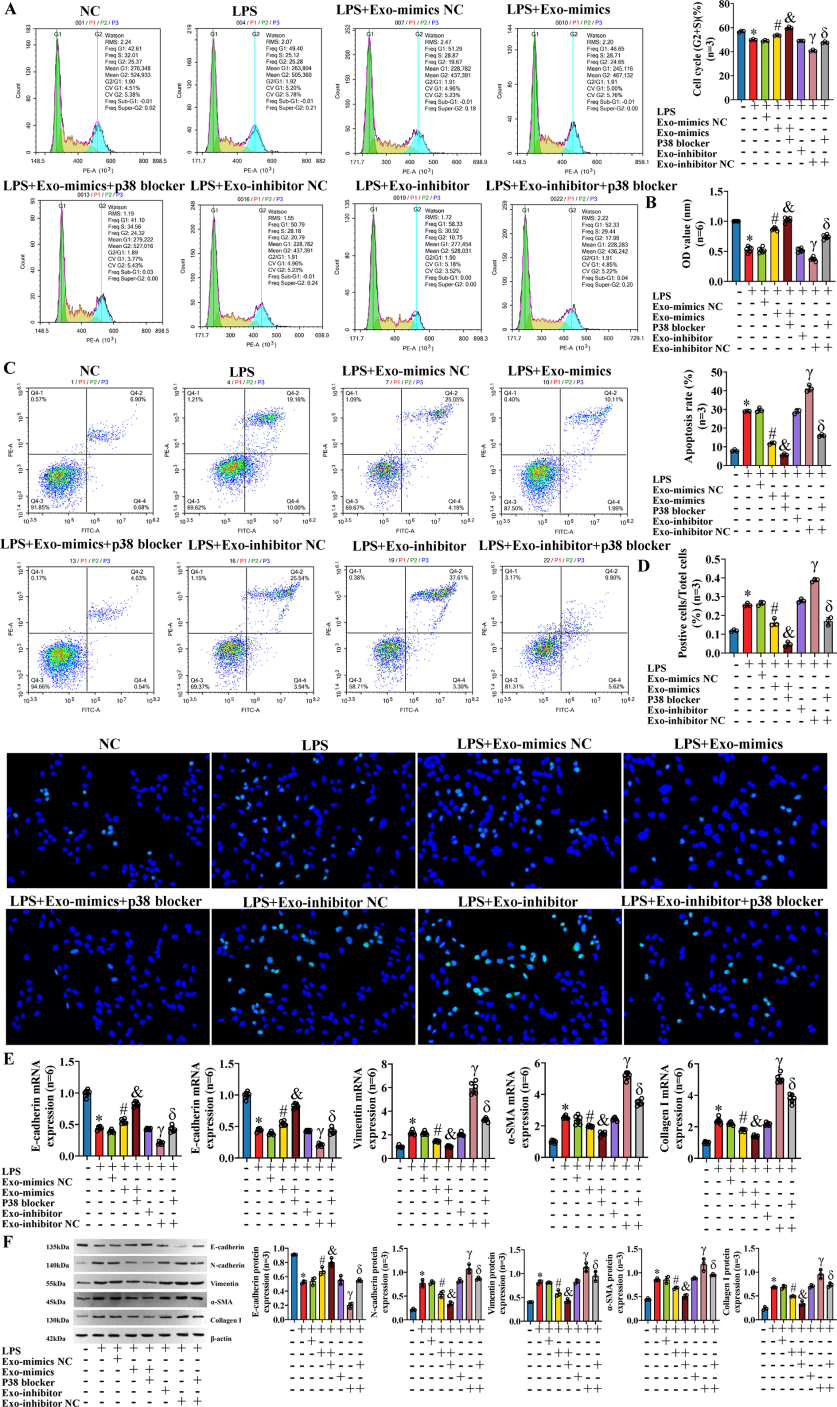
Effects of exosomal miR-122-5p and p38 MAPK pathway blocker on IBECs on the basis of inflammatory model. **(A)** Flow cytometry was used to detect the cycle of IBECs. **(B)** CCK-8 was used to detect the viability of IBECs. **(C, D)** Flow cytometry and TUNEL staining (×200) were used to detect the apoptosis of IBECs. **(E, F)** RT-qPCR and Western blotting were used to assess the mRNA and protein levels of EMT- and fibrosis-related markers in IBECs. *p<0.05, compared to NC group; ^#^ *p*<0.05, compared to LPS+Exo-mimics NC group; ^&^ *p*<0.05, compared to LPS+Exo-mimics group; ^γ^ *p*<0.05, compared to LPS+Exo-inhibitor NC group; ^δ^ *p*<0.05, compared to LPS+Exo-inhibitor group. NC: untreated IBECs; LPS: 0.1 μg/mL LPS treated for 24h; Exo-mimics NC: exosomes transfected with miR-122-5p mimics NC were added to the IBEC culture medium; Exo-mimics: exosomes overexpressing miR-122-5p were added to the IBEC culture medium; p38 blocker: p38 MAPK pathway blocker SB03580 treated for 24h; Exo-inhibitor NC: exosomes transfected with miR-122-5p inhibitor NC were added to the IBEC culture medium; exo-inhibitor: exosomes transfected with miR-122-5p inhibitor were added to the IBEC culture medium. α-SMA, α-smooth muscle actin. Statistical analysis was performed (n ≥ 5) using one-way ANOVA followed by Bonferroni’s *post-hoc* tests; when n < 5, it was performed using permutation tests. IBECs, intrahepatic biliary epithelial cells; CCK-8, Cell Counting Kit-8; EMT, epithelial–mesenchymal transition; LPS, lipopolysaccharide.

To further explore the influence of exosomal miR-122-5p and p38 MAPK pathway blocker on IBECs in the case of inflammatory injury, we co-transfected the IBECs induced by LPS with exosomal miR-122-5p and p38 MAPK pathway blocker. The results showed that in the case of IBEC inflammatory injury, the p38 MAPK pathway blocker also had a synergistic effect with exosomes that overexpressed miR-122-5p and could reverse the effects of LPS and exosomes that had low expression of miR-122-5p on IBECs, and the converse applies. That is, the cycle (G2+S) ratio, cell viability, and E-cadherin were decreased and the apoptosis rate, N-cadherin, vimentin, α-SMA, and collagen I were increased in the LPS group, compared to the IBEC group. The cycle (G2+S) ratio, cell viability, and E-cadherin were increased and the apoptosis rate, N-cadherin, vimentin, α-SMA, and collagen I were decreased in the LPS+Exo-mimics group, compared to the LPS+Exo-mimics NC group. The cycle (G2+S) ratio, cell viability, and E-cadherin were increased and the apoptosis rate, N-cadherin, vimentin, α-SMA, and collagen I were decreased in the LPS+Exo-mimics+p38 blocker group, compared to the LPS+Exo-mimics group. The cycle (G2+S) ratio, cell viability, and E-cadherin were decreased and the apoptosis rate, N-cadherin, vimentin, α-SMA, and collagen I were increased in the LPS+Exo-inhibitor group, compared to the LPS+Exo-inhibitor NC group. The cycle (G2+S) ratio, cell viability, and E-cadherin were increased and the apoptosis rate (**Figures 9A–D**) and N-cadherin, vimentin, α-SMA, and collagen I (**Figures 9E, F**) were decreased in the LPS+Exo-inhibitor+p38 blocker group, compared to the LPS+Exo-inhibitor group (**SI Appendix**, **Supplementary Figures S10**, **S11**).

### 3.6 TNFRSF19 is target gene of miR-122-5p and regulates p38 MAPK pathway

**Figure 10:**
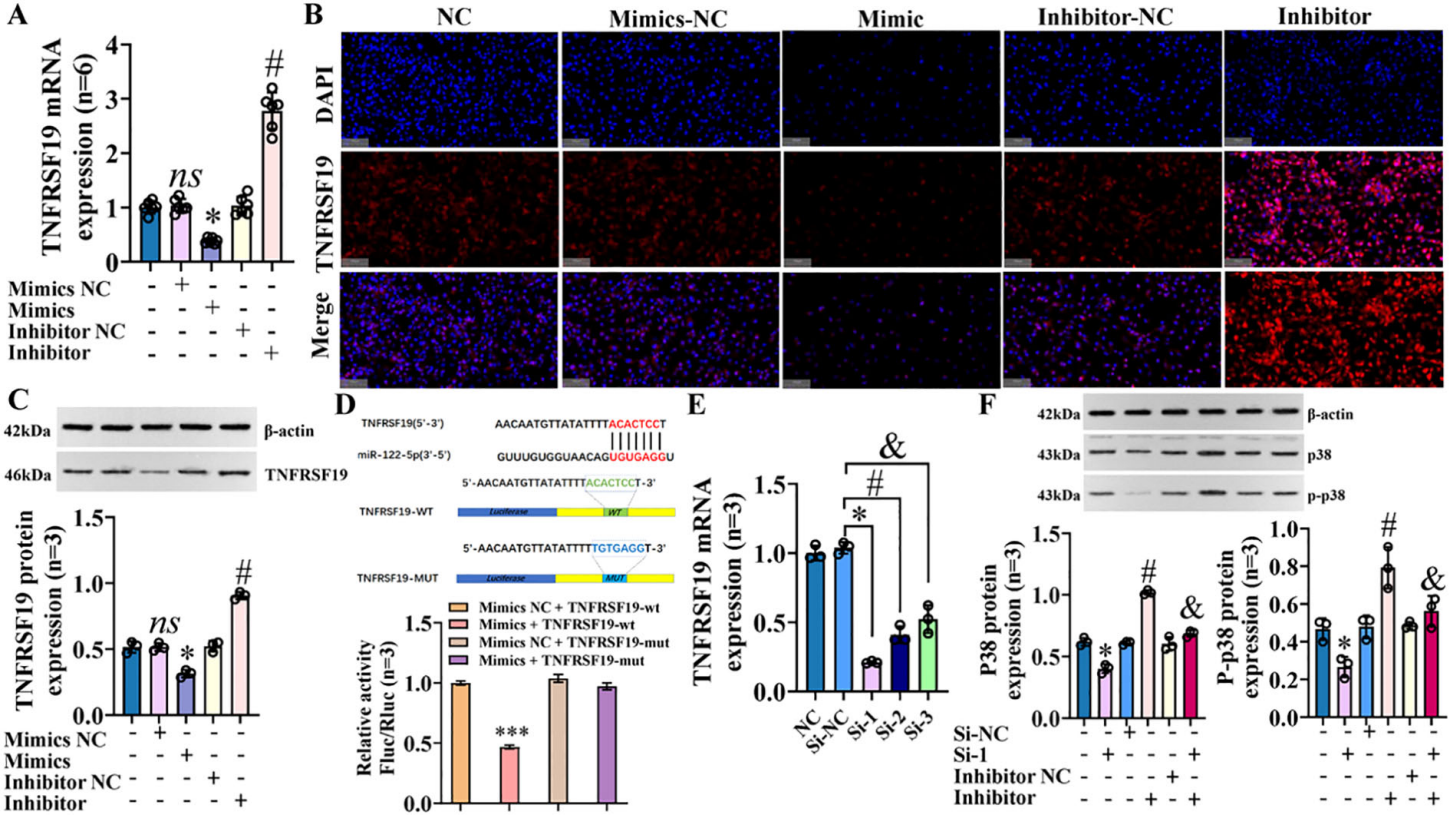
TNFRSF19 is target gene of miR-122-5p and regulates p38 MAPK pathway. **(A)** RT-qPCR was used to detect the expression of TNFRSF19 mRNA; *p<0.05, compared to mimics NC group; *^#^p*<0.05, compared to inhibitor NC group. **(B)** Immunofluorescence was used to detect the expression of TNFRSF19 in IBECs. The nuclei stained by DAPI appear blue, and the positive expression (TNFRSF19) is red when labeled with the corresponding fluorescein. Scale bar, 100 μm. **(C)** Western blotting was used to detect the expression of TNFRSF19 protein. **(D)** Dual luciferase reporter assay proved that TNFRSF is the target gene of miR-122-5p; ***p<0.001, compared with mimics NC+TNFRSF19-wt group. **(E)** TNFRSF19 siRNA screening test; *^#&^ *p*<0.001, compared to si-NC group. **(F)** Western blotting was used to detect the expression of p38 and p-p38 protein; *p<0.05, compared to si-NC; *^#^p*<0.05, compared with inhibitor NC; ^&^ *p*<0.05, compared to inhibitor. Mimics NC and inhibitor NC: negative sequence control of miR-122-5p mimics and inhibitors; mimics and inhibitors: miR-122-5p mimics and inhibitors; si-NC: negative sequence control of TNFRSF19 siRNA; si-1: TNFRSF19 siRNA. Statistical analysis was performed (n ≥ 5) using one-way ANOVA followed by Bonferroni’s *post-hoc* tests; when n < 5, it was performed using permutation tests. IBECs, intrahepatic biliary epithelial cells.

We predicted the target genes of miR-122-5p using TargetScan, miRDB, miRTarBase, and TarBase (**Supplementary Material**—targetgene_query), and we took the intersection with the previous mRNA sequencing results. The Venn plot shows that there are a total of 1,488 overlapping target genes between the two (**SI Appendix**, **Supplementary Figure S12**). Finally, based on the MAPK signaling pathway map (https://www.kegg.jp/pathway/map04010), we speculated that TNFRSF19 may be a target gene of miR-122-5p. To verify this hypothesis, we transfected IBECs with miR-122-5p, which showed that miR-122-5p negatively regulated the expression of TNFRSF19 (**Figures 10A–C**) (**SI Appendix**, **Supplementary Figure S13A**). Dual luciferase reporter assay showed that the relative luciferase activity in the TNFRSF19-wt+mimics group was significantly lower than that in the TNFRSF19-wt+mimics NC group (**Figure 10D**). This indicated that TNFRSF19 had binding sites with miR-122-5p and was the target gene of miR-122-5p.

To verify that TNFRSF19 was targeted by miR-122-5p and then regulates the p38 MAPK signaling pathway, we designed three different siRNAs and selected siRNA-1 (TNFRSF19-785, 79.7%) with the highest interference efficiency by detecting the level of TNFRSF19 mRNA to complete the following tests (**Figure 10E**). The transfection of TNFRSF19 siRNA (si-1 for short) resulted in a significant decrease in the expression of p38 and p-p38 proteins, indicating that TNFRSF19 regulates the p38 MAPK signaling pathway. Moreover, the protein expression of p38 and p-p38 was decreased in the inhibitor+si-1 group (**Figure 10F**) compared to the inhibitor group (**SI Appendix**, **Supplementary Figure S13B**). This means that TNFRSF19 siRNA can reverse the increase in p38 and p-p38 protein caused by the low expression of miR-122-5p. That is, miR-122-5p really regulates the p38 MAPK signaling pathway by targeting TNFRSF19. In summary, miR-122-5p targeting TNFRSF19 affects the proliferation and apoptosis of IBECs, EMT, and fibrosis via the p38 MAPK signaling pathway.

### 3.7 TNFRSF19 regulates p38 MAPK pathway through ASK1

**Figure 11:**
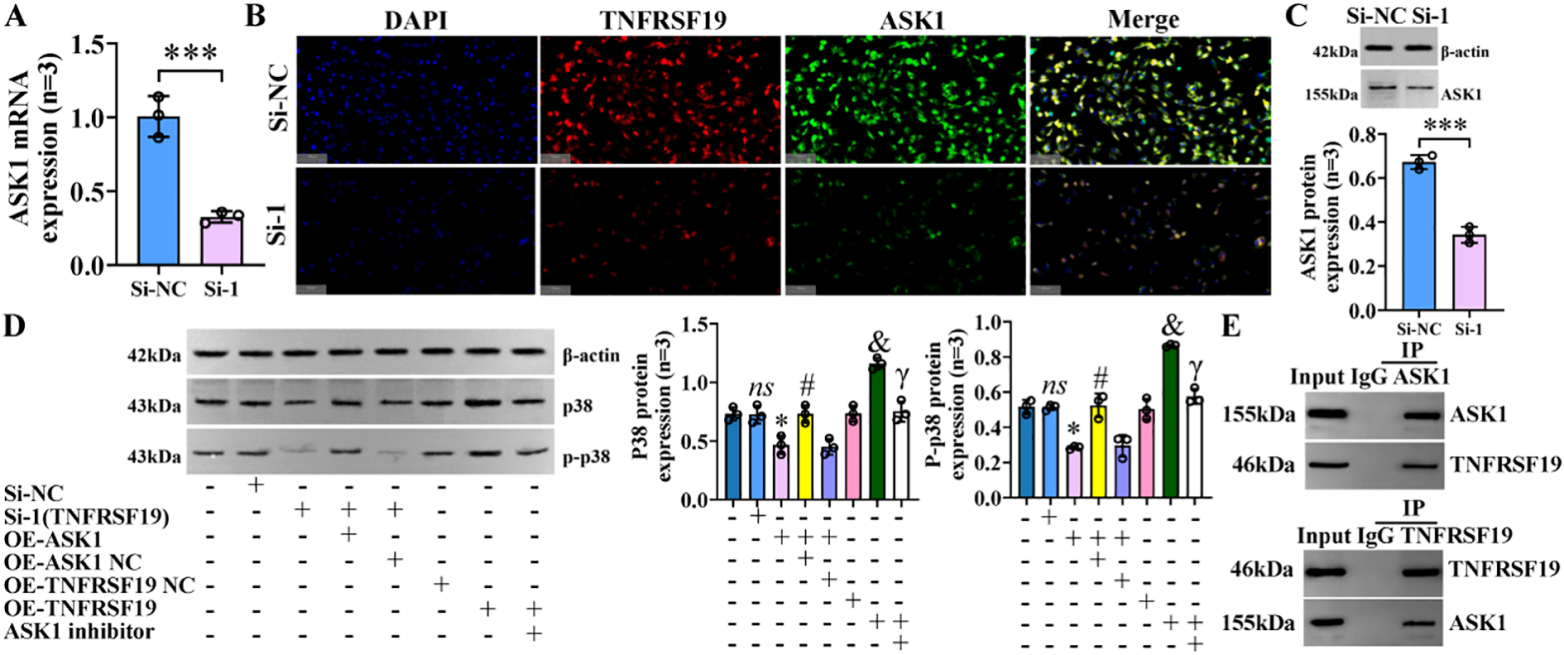
TNFRSF19 regulates p38 MAPK signaling pathway through ASK1. **(A)** TNFRSF19 siRNA downregulates ASK1 mRNA levels; ***p<0.001, compared to si-NC. **(B)** Immunofluorescence was used to detect the expression of TNFRSF19 and ASK1 in IBECs. The nuclei stained by DAPI appear blue, and the positive expression is red (TNFRSF19) and green (ASK1) when labeled with the corresponding fluorescein. Scale bar, 100 μm. **(C)** TNFRSF19 siRNA downregulates ASK1 protein levels. **(D)** Overexpression of ASK1 can reverse the effects of si-TNFRSF19 on p38 and p-p38 protein levels. **(E)** The Co-IP experiment confirmed the interaction between TNFRSF19 and ASK1. *ns*, no significance; *p<0.05, compared to si-NC; ^#^ *p*<0.05, compared to si-TNFRSF19+OE-ASK1 NC group; ^&^ *p*<0.05, compared to OE-TNFRSF19 NC group; ^γ^ *p*<0.05, compared to OE-TNFRSF19 group. Si-NC: negative sequence control of TNFRSF19 siRNA; si-1: TNFRSF19 siRNA; OE-ASK1: overexpression of ASK1 plasmid; OE-ASK1 NC: negative sequence control of ASK1 plasmid; OE-TNFRSF19: overexpression of the TNFRSF19 plasmid; OE-TNFRSF19 NC: negative sequence control of the TNFRSF19 plasmid. Statistical analysis was performed using Student’s t-test and one-way ANOVA, followed by Bonferroni’s *post-hoc* tests; when n < 5, it was performed using permutation tests. IBECs, intrahepatic biliary epithelial cells; Co-IP, co-immunoprecipitation.

ASK1 is known to be the upstream regulatory protein of the p38 MAPK signaling pathway, which can be stimulated and activated by various substances, including stress and cytokines such as TNF-α (20). We further explored whether TNFRSF19 also regulates the p38 MAPK signaling pathway through ASK1. Pre-experimental results showed that the level of ASK1 was decreased in the si-1 group, compared to the si-NC group (**Figures 11A–C**). It means that TNFRSF19 could regulate ASK1. In the formal experiment, we transfected TNFRSF19 siRNA, plasmid (OE-TNFRSF19 and OE-ASK1), control (si-NC, OE-TNFRSF19 NC, and OE-ASK1 NC), and ASK1 inhibitor. The results showed that the protein levels of p38 and p-p38 in the si-1+OE-ASK1 group were higher than those in the si-1+OE-ASK1 NC group. The protein levels of p38 and p-p38 in the OE-TNFRSF19+ASK1 inhibitor group were higher than those in the OE-TNFRSF19 group (**Figures 11D, E**) (**SI Appendix**, **Supplementary Figure S14**). This result further proves that TNFRSF19 regulates the p38 MAPK signaling pathway through ASK1.

### 3.8 The exosomes derived from HSCs deliver miR-122-5p to alleviate the pathological damage of liver tissue

**Figure 12:**
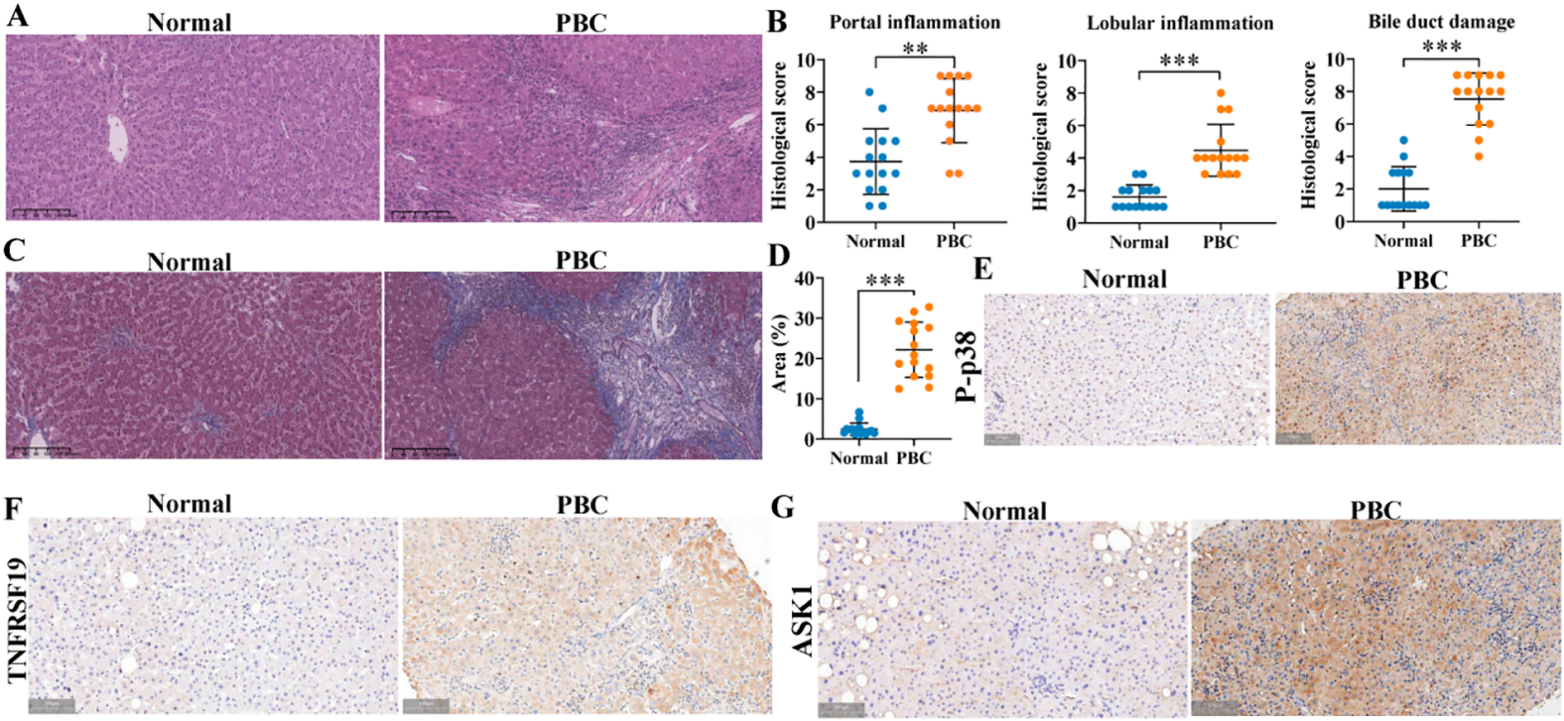
Pathological staining of liver tissue in clinical subjects. **(A, B)** H&E staining and pathological score. Scale bar, 200 μm. **(C, D)** Masson staining and collagen area. Normal: liver tissue structure was normal, and there was no obvious degeneration or necrosis of liver cells. Scale bar, 200 μm. A small amount of inflammatory cells could be seen in some of the inlet areas, no obvious inflammation was seen in the other lobules and the inlet areas, and no bile duct damage was seen. PBC: more liver cells in the lobule with mild vesicular lipolysis, spotty necrosis, a few small fragments had necrosis, Kupffer cell proliferation, and mild to moderate inter-facial inflammation; the portal area was enlarged, interlobular bile duct was destroyed more with medium to multiple inflammatory cell infiltration, fibrous tissue hyperplasia, and interlobular fibrous septa was formed in some areas. **(E–G)** Immunohistochemical staining was used to detect the expression of p-p38, TNFRSF19, and ASK1. Brown indicates the position of positive expression. Scale bar, 100 μm. **p<0.01, ***p<0.001, compared to normal group. α-SMA, α-smooth muscle actin. Statistical analysis was performed using Student’s t-test. PBC, primary biliary cholangitis.

**Figure 13:**
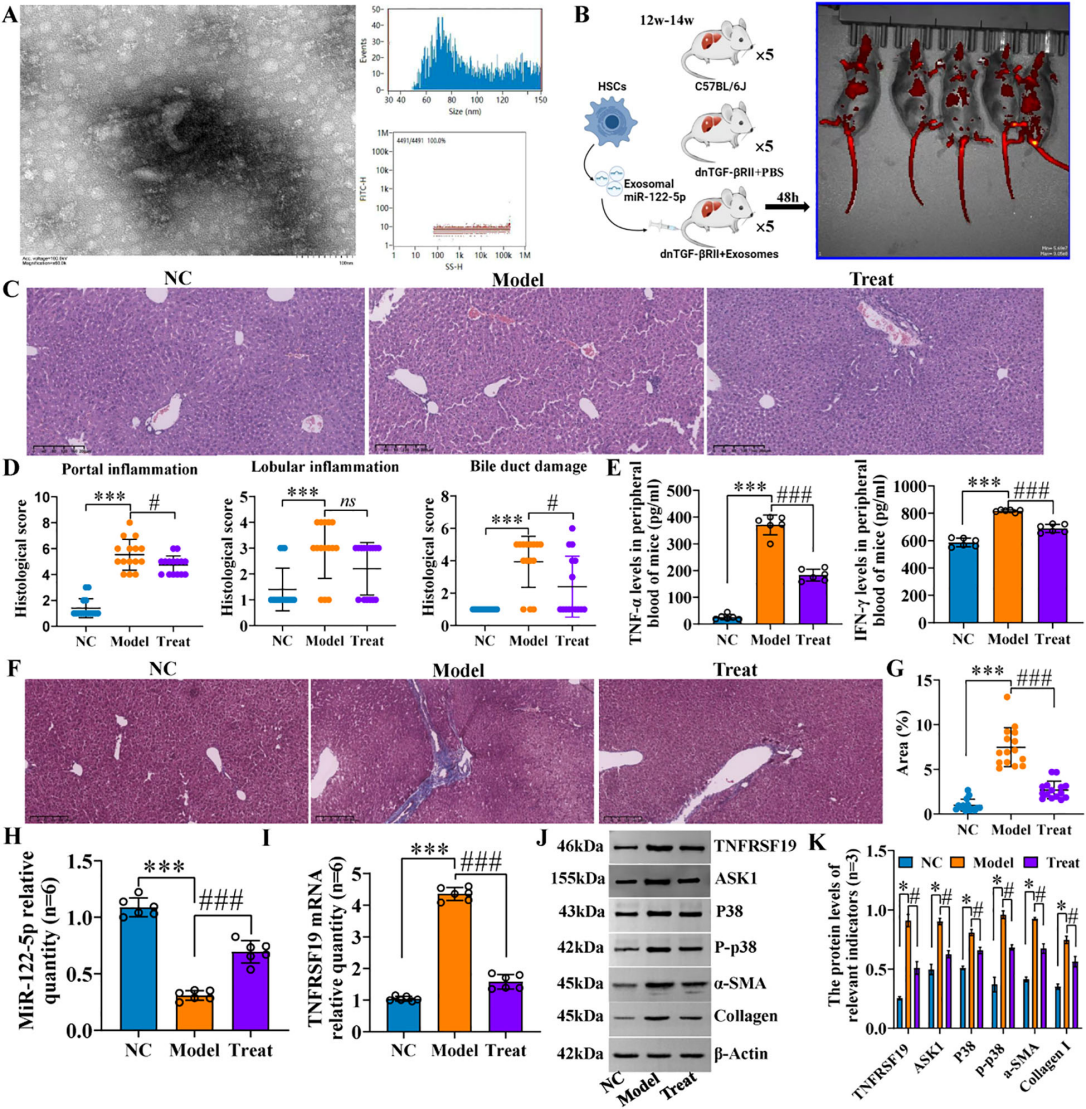
Hepatic portal inflammation and bile duct damage of PBC model mice were alleviated after exosome treatment. **(A)** Identification of exosomes from mouse HSCs. Through transmission electron microscopy and particle size detection, the identification results were consistent with the characteristics of exosomes: the exosomes captured under the transmission electron microscope are small, round, or oval vesicles with a shape similar to “tea tray type” or concave hemispherical type, and have a distinct bilayer membrane structure. The particle sizes were evenly distributed in the range of 40–150 nm, with an average particle size of 81.68 nm and a concentration of 8.16 × 10^8^ particles/mL. **(B)** Flowchart of animal experiment and *in vivo* imaging. **(C, D)** H&E staining and pathological score. **(E)** Inflammatory factor levels in peripheral blood. Scale bar, 200 μm. **(F, G)** Masson staining and collagen area. Scale bar, 200 μm. NC (C57BL/6J): liver cell structure was normal, no obvious inflammation was observed in the lobule and the portal area, no bile duct damage, and no fiber tissue proliferation was observed in the lobule and the portal area. Model (dnTGF-βRII+PBS): scattered spot-like necrosis of hepatic cells in the lobules, individual small fragmentary necrosis, slight increase in Kupffer cells, mild to moderate inflammation in the local portal area (mainly lymphocyte infiltration), disappearance of local interlobular bile duct destruction with lymphocyte infiltration (that is, the specific manifestation of chronic non-suppurative destructive cholangitis), and obvious proliferation of fibrous tissue in the portal area. Treat (dnTGF-βRII+exosomes): mild alveolar steatosis of some lobules of hepatocytes, very individual spot-like necrosis, slight increase in Kupffer cells, a small amount of inflammatory cell infiltration in the local area, no obvious bile duct damage, mild hyperplasia of the local small bile duct, and a small amount of fibrous hyperplasia in the portal area. ***p<0.001, compared to NC group; ^###^ *p*<0.001, compared to Model group; *p<0.01, compared to Model group; ns, no significance. **(H, I)** RT-qPCR was used to detect the levels of miR-122-5p and TNFRSF19 mRNA in the three groups of animals. **(J, K)** Western blotting was used to detect the protein levels of NFRSF19, ASK1, p38 pathways, and fibrosis indicators in the three groups of animals.*p<0.001, compared to NC group; ^###^ *p*<0.001, compared to Model group. α-SMA, α-smooth muscle actin. Statistical analysis was performed using one-way ANOVA followed by Bonferroni’s *post-hoc* tests. PBC, primary biliary cholangitis; HSCs, hepatic stellate cells.

We identified mouse genes using agarose gel electrophoresis (**SI Appendix**, **Supplementary Figure S15**) and then evaluated hepatic inflammation and fibrosis using H&E and Masson staining. The results revealed that the hepatic portal and lobular inflammation, bile duct damage, and fibrosis (**Figures 12A–D**) of clinical PBC patients were more serious than those of normal controls. Immunohistochemical staining indicated that the levels of p-p38, TNFRSF19, and ASK1 were higher than those of normal controls (**Figures 12E–G**). Our previous studies (11) have proved that exosomal miR-122-5p is derived from HSCs. To further verify the effect of exosomal miR-122-5p on liver tissues of PBC patients, we transferred mmu-miR-122-5p into mouse HSCs and then extracted exosomes. The identification results of exosomes are shown in **Figure 13A**. Exosomes were labeled with PKH26. *In vivo* imaging of mice and liver RT-qPCR indicated that exosomes carrying miR-122-5p reached the liver (**Figures 13B, H**, **SI Appendix**, **Supplementary Figure S16**). The results showed that PBC model mice had hepatic portal, lobular inflammation, bile duct damage, and fibrosis and that the levels of TNF-α and TNF-γ in peripheral blood were more serious than those of normal control mice. The hepatic portal inflammation, bile duct damage, and fibrosis of model mice injected with exosomal miR-122-5p were less severe than those of non-injected model mice, and the levels of TNF-α and TNF-γ in peripheral blood also decreased (**Figures 13C–G**). In addition, we further detected TNFRSF19 and fibrosis index in the liver tissues of three groups of mice. The results showed that the levels of TNFRSF19, fibrosis index α-SMA, and collagen I in the liver tissue of PBC model mice were higher than those of control mice. After injection of exosomes with overexpressed miR-122-5p, the levels of TNFRSF19, α-SMA, and collagen I were decreased compared with those of non-injected model mice (**Figures 13I–K**).

## 4 Discussion

Our previous studies have confirmed that exosomal miR-122-5p is derived from HSCs and regulates the expression of IBEC inflammatory factors through the p38 MAPK signaling pathway (11). In this study, we found that exosomal miR-122-5p is associated with liver injury and cholestasis indicators, and the combination of gp210 and sp100 antibodies can improve the sensitivity of PBC diagnosis. *In vitro* experiments have shown that exosome miR-122-5p regulates ASK1 level by targeting TNFRSF19, promotes IBEC proliferation through the p38 MAPK signaling pathway, and inhibits apoptosis, EMT, and fibrosis. Animal experiments further confirmed that the exosomal miR-122-5p can improve the pathological damage of liver tissue in PBC model mice.

Exosomes are known to be important communication mediators between cells, mediating miRNAs to participate in various life processes in the body, and do not increase the risk of tumorigenesis or immune response (21, 22). Our previous study found that the expression of miR-122-5p in serum exosomes of PBC patients was increased (11). MiR-122-5p is a highly abundant liver-specific miRNA (23), which has been identified as a marker of various liver injuries (24–28). In this study, we analyzed the correlation between exosomal miR-122-5p and liver function, and we found that exosomal miR-122-5p was positively correlated with ALT, AST, DBIL, γ-GT, ALP, and TBA, but had no significant correlation with ALB, TBIL, and IBIL. This may be related to the characteristics of biochemical indices in PBC patients; that is, the increase in DBIL, γ-GT, ALP, ALT, AST, and TBA was evident in PBC, while the increase in TBIL and IBIL was not obvious, and the level of ALB did not change in the early stage. In addition, the ROC curve showed that the combination of exosomal miR-122-5p with sp100 and gp210 antibodies can improve the sensitivity of diagnosing PBC. That is, when clinical patients have positive autoantibodies, normal liver function indicators, or abnormal liver function indicators but negative AMA-M2 and are unwilling to undergo liver biopsy, the level of miR-122-5p in serum exosomes can be combined to assist in clinical diagnosis. Therefore, exosomal miR-122–5 is expected to be a potential biomarker of PBC.

Studies have reported that mice with miR-122/miR-122-5p gene deletion show a high incidence of lipid metabolism alteration, liver inflammation, liver spontaneous fibrosis, and hepatocellular carcinoma (13, 14). Li et al. (29) showed that miR-122-5p could inhibit the proliferation of activated HSC-LX2 and reduce collagen maturation by targeting P4HA1, thus inhibiting liver fibrosis. The inhibition of miR-122-5p expression resulted in increased collagen maturation and extracellular matrix production, which is basically consistent with the results of this study. However, current studies on miR-122-5p and liver fibrosis mainly focus on HSCs. It is known that IBECs are the main target cells of early PBC injury, and bile duct proliferation and disappearance are the main causes of cholestatic cirrhosis (30). However, there have been few reports on miR-122-5p and IBECs so far. Our previous studies have shown that exosomes derived from HSCs mediate miR-122-5p to regulate the expression of IBEC inflammatory factors through the p38 MAPK signaling pathway (11). In this study, exosomes overexpressing miR-122-5p were transfected into human IBECs, and LPS-induced inflammatory damage of IBECs was used to simulate the state of IBECs in PBC patients. The results showed that the overexpression of miR-122-5p in exosomes promoted the proliferation of IBECs and inhibited the apoptosis, EMT, and fibrosis of IBECs; the effect was synergistic after blocking the p38 MAPK signaling pathway. On the contrary, low expression of miR-122-5p in exosomes inhibited the proliferation of IBECs and promoted the apoptosis, EMT, and fibrosis of IBECs. This effect was reversed after blocking the p38 MAPK signaling pathway. These indicated that exosomal miR-122-5p could also promote the proliferation of IBECs and inhibit the apoptosis, EMT, and fibrosis of IBECs through the p38 MAPK signaling pathway.

Under normal conditions, cell proliferation and apoptosis maintain a dynamic balance. Apoptosis is essential for the maintenance of immune cell populations (31, 32). Some scholars have studied the apoptosis of biliary epithelial cells in PBC, and their results showed that DNA fragments were increased in biliary epithelial cells in PBC patients and that the levels of apoptosis-inducing ligands associated with Fas, FasL, perforin, granzyme B, and TNF were significantly increased (33–35). In addition, compared with other chronic cholestasis diseases with similar levels of inflammation, biliary epithelial cell apoptosis is increased in PBC patients (33–37). EMT refers to the biological process by which epithelial cells are transformed into cells with an interstitial phenotype by a specific procedure. It plays an important role in chronic inflammation, tissue reconstruction, cancer metastasis, and a variety of fibrosis diseases (38, 39). Its main characteristics include reduction/loss of E-cadherin and upregulation of N-cadherin and vimentin, which contribute to the fibrogenesis process (40, 41). Immunohistochemistry studies of liver sections from patients with PBC showed attenuated epithelial markers and increased mesenchymal markers in cholangiocytes (9). A study of patients with PBC after liver transplantation found that EMT occurs before the development of any other features of recurrent PBC, suggesting that EMT may be an initiating event (potentially explaining biliary epithelial cells loss) (42). Therefore, the inhibitory effect of miR-122-5p on IBEC apoptosis, EMT, and fibrosis may have certain prospects in the treatment of PBC.

TNFRSF19 is an orphan member of the TNF receptor superfamily, and most previous studies on TNFRSF19 focused on tumor-related diseases (43, 44). In recent years, TNFRSF19 has been found to be involved in cytokine and cytokine receptor interactions and associated with B-cell survival (45, 46). ASK1, also known as MAP3K5, is a widely expressed member of the MAPK family, regulating the JNK and p38 MAPK signaling pathways (47). ASK1 is expressed in a variety of tissues and is involved in apoptosis, inflammation, oxidative stress, and other processes. The abnormal activation of ASK1 can lead to pathological changes in these tissues (48–50). In the liver, the abnormal activation of ASK1 can lead to a variety of liver diseases, such as fatty liver (51), liver fibrosis, and liver cancer (52, 53). In this study, we found that TNFRSF19 is a target gene of miR-122-5p and can regulate the level of ASK1, and the overexpression of ASK1 can reverse the decrease in p38 and p-p38 caused by TNFRSF19 siRNA. These indicate that TNFRSF19 can regulate the p38 MAPK signaling pathway through ASK1. To our knowledge, this is the first study to report the molecular mechanism of exosomal miR-122-5p and PBC.

**Figure 14:**
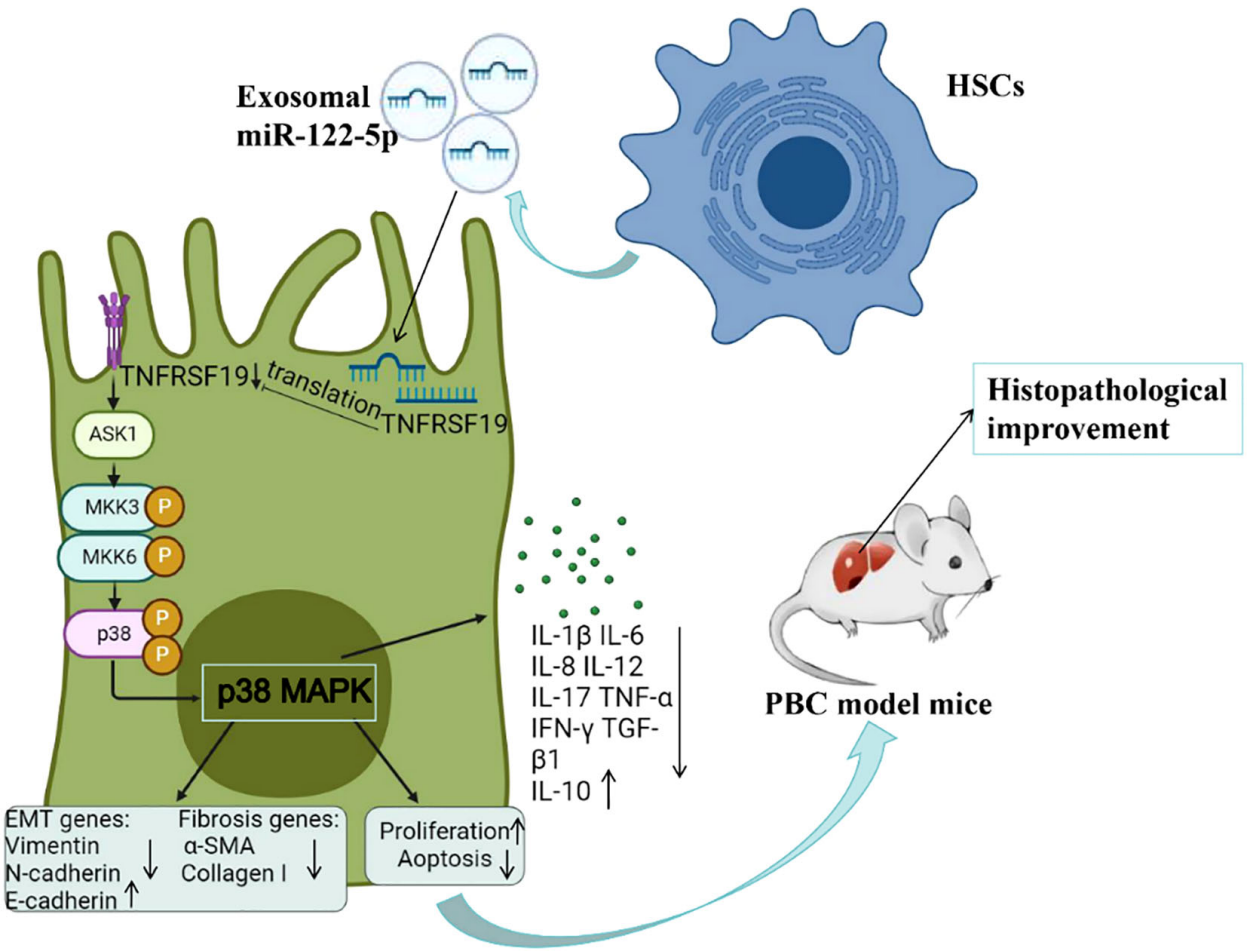
Molecular mechanism diagram of exosomal miR-122-5p alleviating liver tissue pathological damage. PBC, primary biliary cholangitis; HSCs, hepatic stellate cells; α-SMA, α-smooth muscle actin; ASK1, apoptosis signal-regulated kinase 1; TNFRSF19, tumor necrosis factor receptor superfamily 19.

In order to further investigate the effect of exosomal miR-122-5p on the pathological damage of PBC, we injected exosomes overexpressing miR-122-5p into PBC model mice (dnTGF-βRII) through the tail vein and compared the inflammation of the hepatic portal and lobule, bile duct damage, and fibrosis by H&E and Masson staining. The results showed that the hepatic portal inflammation, bile duct damage, and fibrosis of the PBC model mice were less severe than those of non-injected model mice, and the levels of TNF-α and TNF-γ in peripheral blood also decreased. Lobular inflammation of liver tissue was slightly improved but not statistically significant, which may be related to the characteristics of dnTGF-βRII (lobular inflammation itself is not serious). In addition, the TNFRSF19, ASK1, p-p38, and fibrosis indices in liver tissue were basically consistent with the cellular results, indicating that exosomal miR-122-5p does indeed target TNFRSF19 to regulate the ASK1 level and alleviate the pathological damage of liver tissue in PBC mice via the p38MAPK signaling pathway (**Figure 14**). Meanwhile, this study also has some limitations. For instance, the combined AUC results of this study have not yet been verified in independent cohorts, which is a work that needs to be carried out in the future. In addition, the sample size was relatively small, with only one male patient, making it impossible to analyze the gender differences in PBC. Due to the relatively small number of patients undergoing liver puncture, it is impossible to analyze the level of miR-122-5p based on the severity of the pathology. Moreover, only one PBC model mouse was included in this study. In subsequent studies, other PBC models (such as Il2ra^−/−^ mice) can be considered to verify the role of exosome miR-122-5p in PBC more comprehensively.

In summary, this study is the first to explore the relationship between serum exosomal miR-122-5p and liver function and diagnostic antibodies in patients with PBC. Through *in vivo* and *in vitro* experiments, it was further confirmed that exosomal miR-122-5p affected the level of ASK1 by targeting TNFRSF19, regulated the p38 MAPK signaling pathway, and inhibited the apoptosis, EMT, and fibrosis of IBECs, thus alleviating the pathological damage of PBC liver tissue. Therefore, we believe that the exosomal miR-122-5p is expected to be a potential marker and therapeutic target for the diagnosis of PBC.

## Data Availability

The datasets presented in this study can be found in online repositories. The names of the repository/repositories and accession number(s) can be found below: https://www.ncbi.nlm.nih.gov/, GSE231432.
