# Supplementary material for: HSC-derived exosomal miR-122-5p inhibits EMT and fibrosis of intrahepatic biliary epithelial cells to alleviate primary biliary cholangitis
Source: Front Immunol. 2025 Oct 31;16:1684064. doi: 10.3389/fimmu.2025.1684064 (PMC12615470; doi:10.3389/fimmu.2025.1684064)
Supplement: Supplementary file 2 [file Table1.docx]

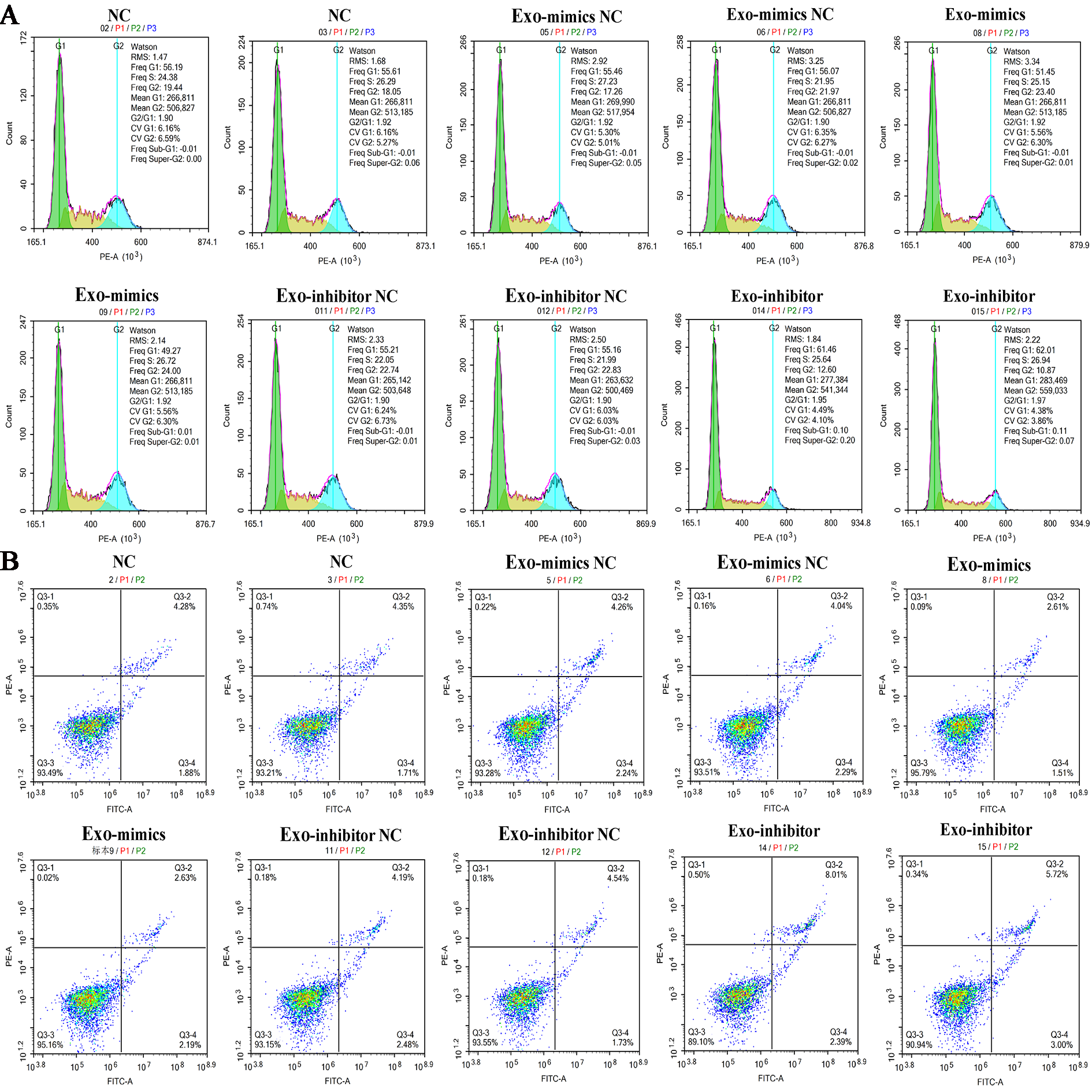


**Figure S1. Exosomal miR-122-5p promoted the proliferation, and inhibited the apoptosis**


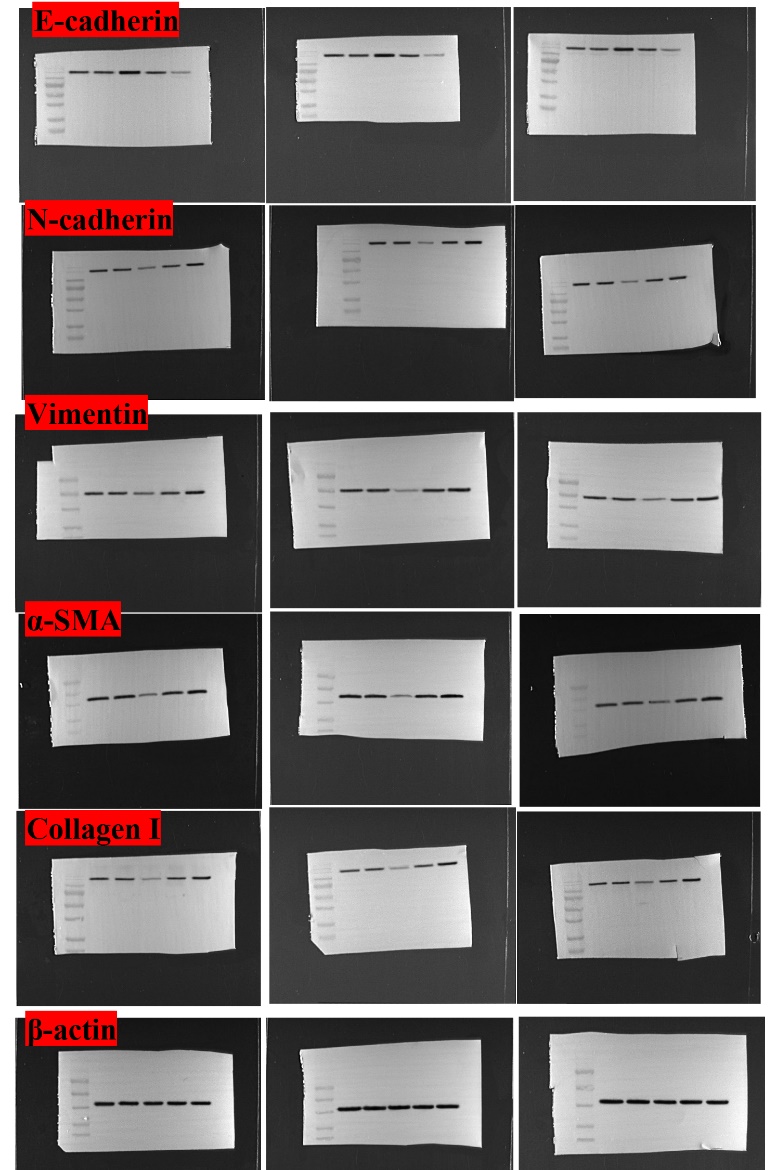


**25kDa**

**35kDa**

**70kDa**

**55kDa**

**40kDa**

**25kDa**

**180kDa**

**100kDa**

**35kDa**

**55kDa**

**130kDa**

**40kDa**

**70kDa**

**70kDa**

**35kDa**

**55kDa**

**40kDa**

**25kDa**

**130kDa**

**45kDa**

**42kDa**

**55kDa**

**70kDa**

**40kDa**

**35kDa**

**25kDa**

**25kDa**

**55kDa**

**130kDa**

**140kDa**

**100kDa**

**70kDa**

**180kDa**

**35kDa**

**40kDa**

**100kDa**

**180kDa**

**35kDa**

**25kDa**

**40kDa**

**55kDa**

**130kDa**

**70kDa**

**55kDa**

**135kDa**

**Figure S2. Exosomal miR-122-5p inhibited EMT and fibrosis of human IBECs (full uncropped western blots). IBECs: intrahepatic biliary epithelial cells**

**
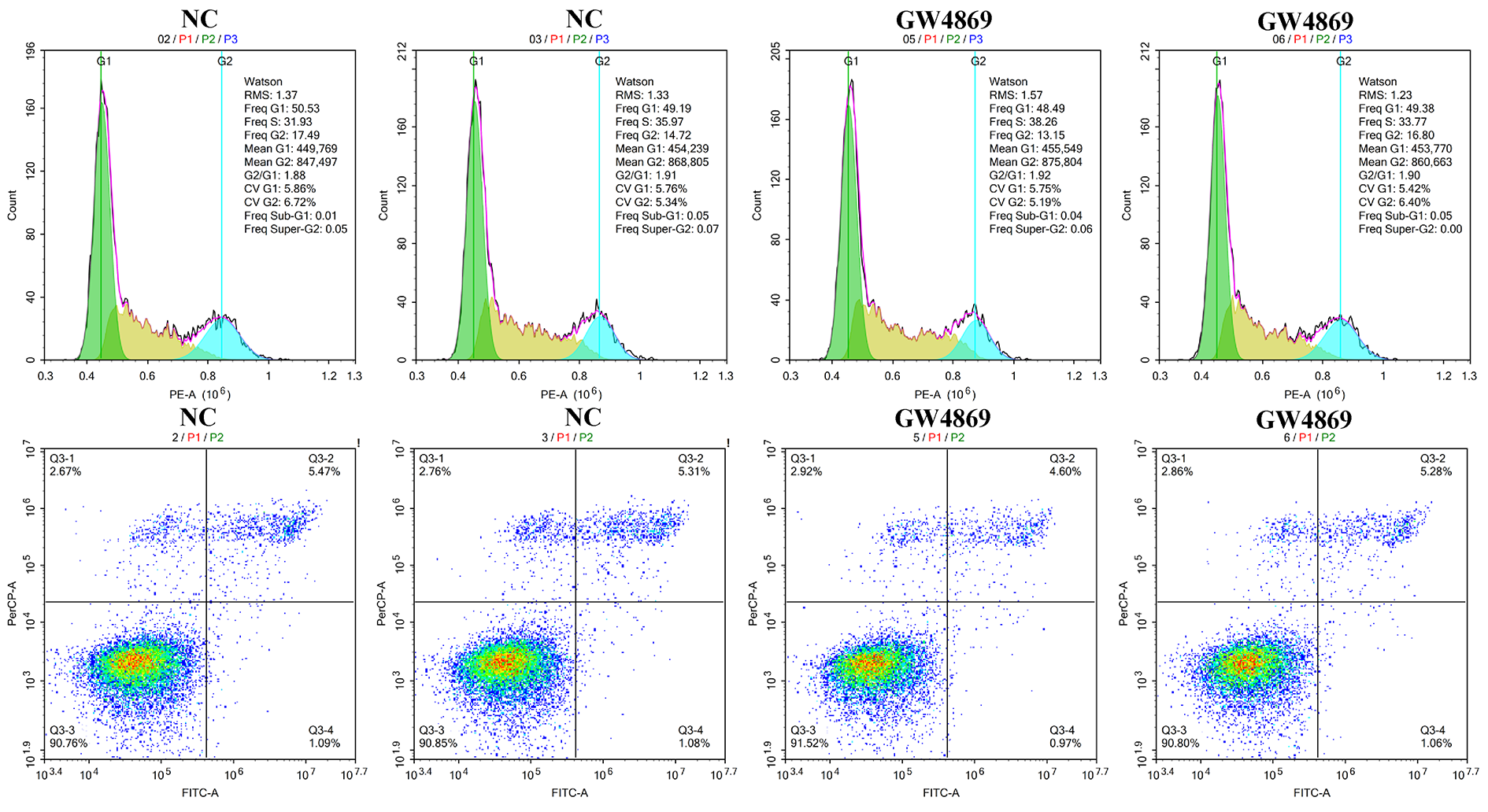
**

**
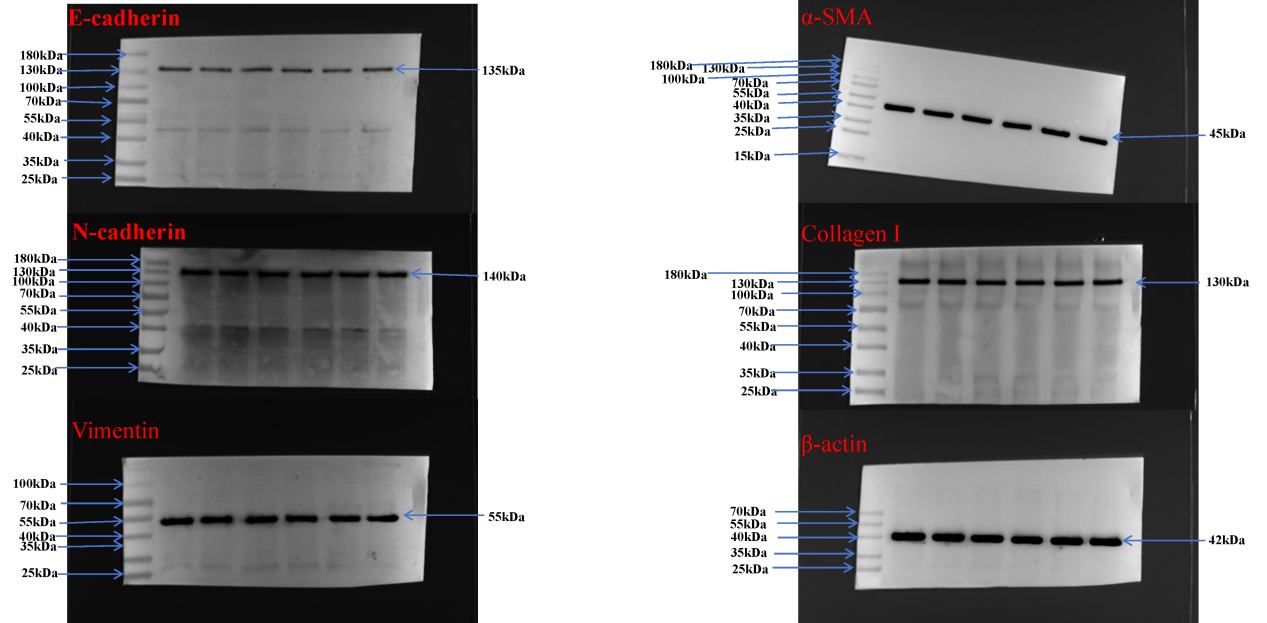
**

**Figure S3. Exosomes have no effect on the phenotypic changes of IBECs**

**
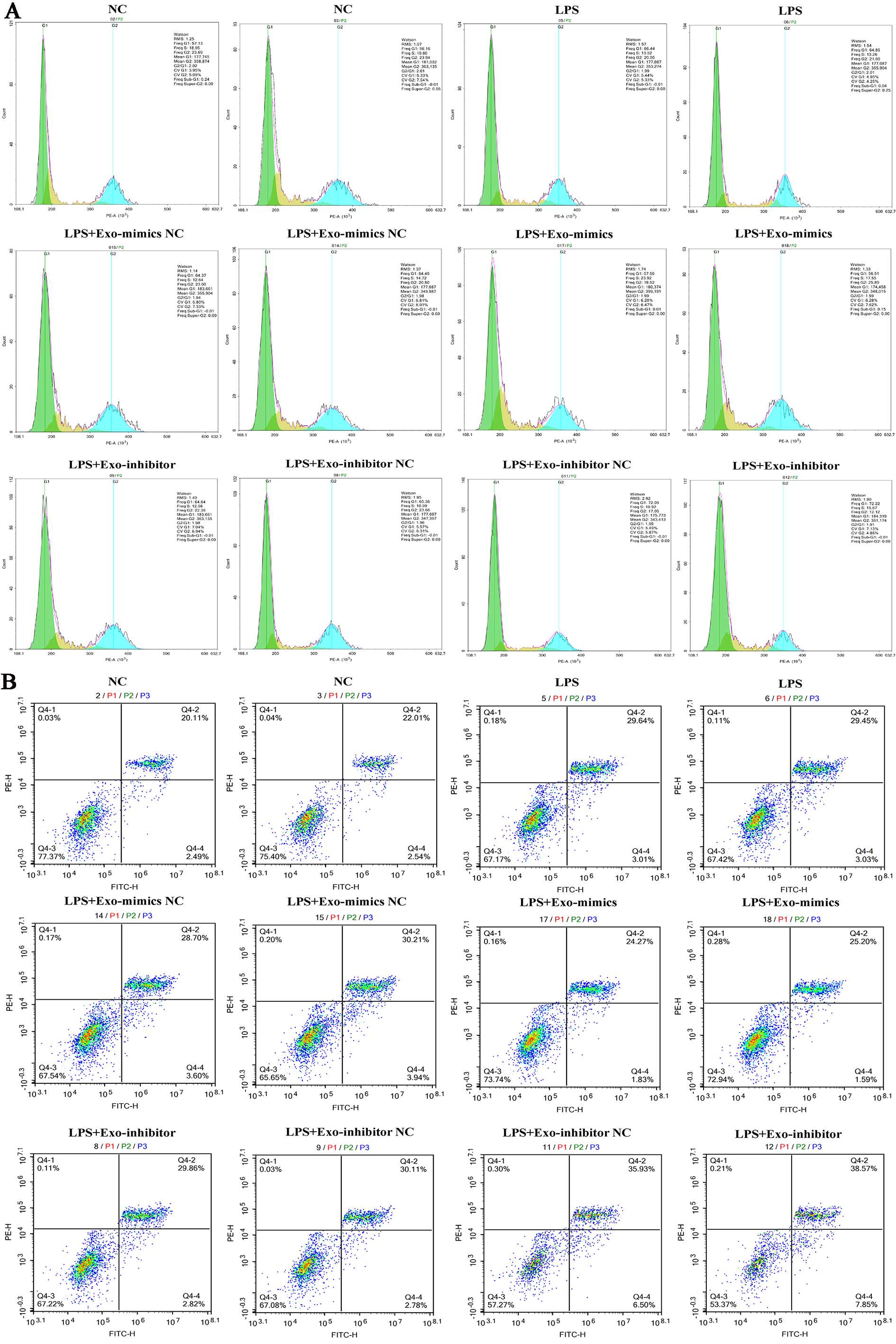
**

**Figure S4. The effect on human IBECs of LPS was reversed by exosomal miR-122-5p.IBECs: intrahepatic biliary epithelial cells**


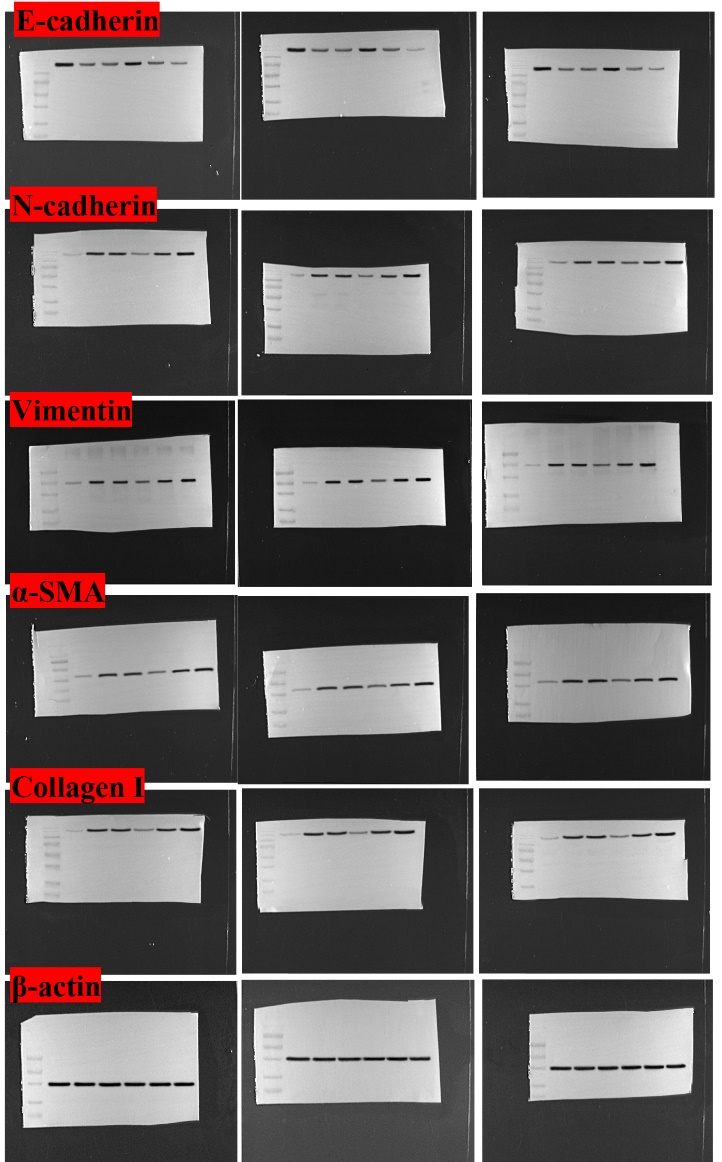


**25kDa**

**35kDa**

**40kDa**

**70kDa**

**55kDa**

**25kDa**

**35kDa**

**40kDa**

**70kDa**

**55kDa**

**25kDa**

**35kDa**

**40kDa**

**55kDa**

**70kDa**

**100kDa**

**130kDa**

**180kDa**

**25kDa**

**35kDa**

**70kDa**

**55kDa**

**40kDa**

**25kDa**

**35kDa**

**40kDa**

**55kDa**

**70kDa**

**130kDa**

**100kDa**

**180kDa**

**25kDa**

**35kDa**

**40kDa**

**55kDa**

**70kDa**

**100kDa**

**130kDa**

**180kDa**

**42kDa**

**130kDa**

**55kDa**

**140kDa**

**135kDa**

**45kDa**

**Figure S5. Exosomal miR-122-5p reverses the effect of LPS in promoting EMT and fibrosis in human IBECs (full uncropped western blots). IBECs: intrahepatic biliary epithelial cells**

**
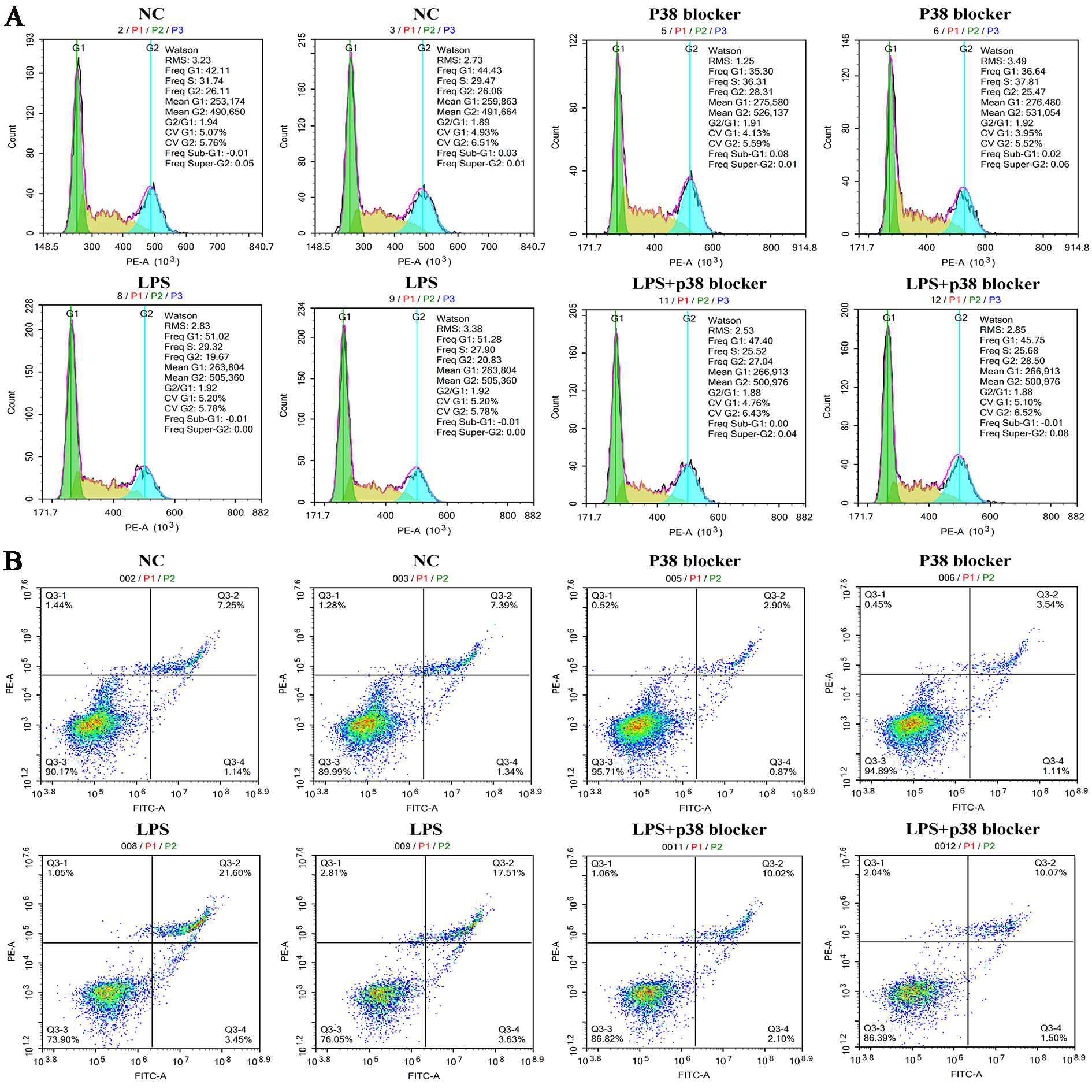
**

**Figure S6. The proliferation of human IBECs increased and apoptosis decreased after**

**the p38 MAPK signaling pathway was blocked.**


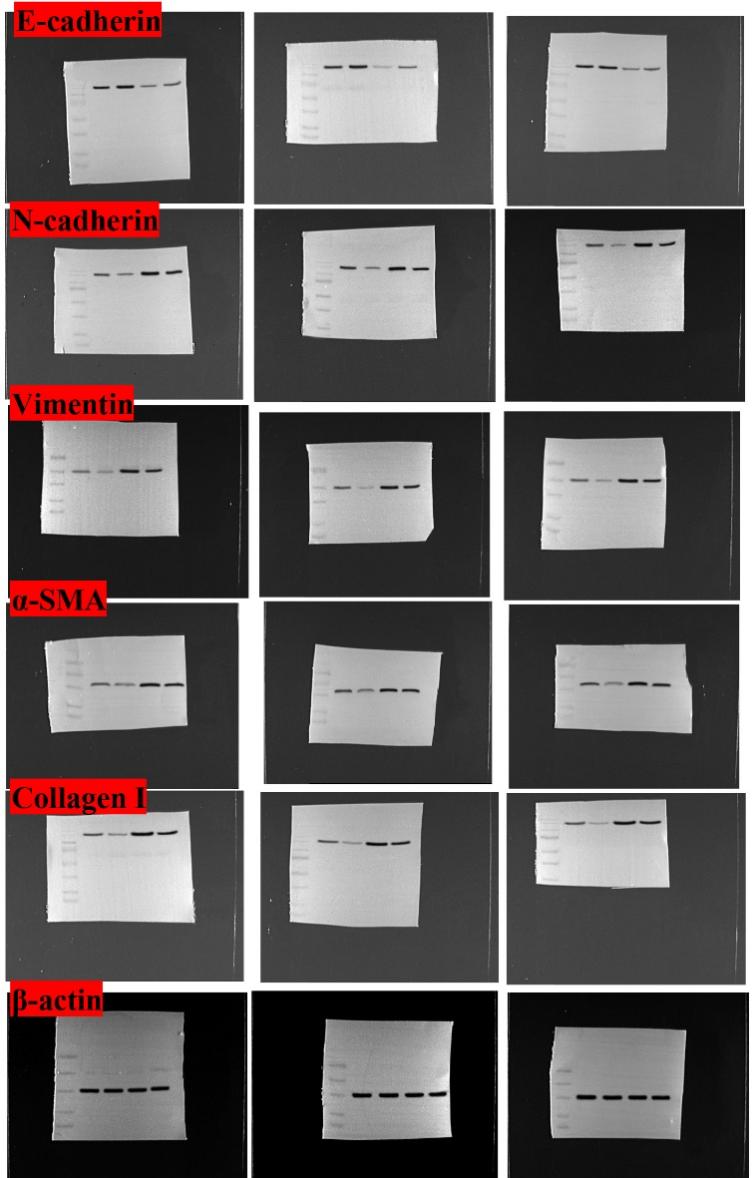


**25kDa**

**35kDa**

**40kDa**

**55kDa**

**70kDa**

**25kDa**

**35kDa**

**40kDa**

**55kDa**

**70kDa**

**100kDa**

**130kDa**

**180kDa**

**42kDa**

**130kDa**

**45kDa**

**55kDa**

**140kDa**

**135kDa**

**25kDa**

**35kDa**

**40kDa**

**55kDa**

**70kDa**

**25kDa**

**35kDa**

**40kDa**

**70kDa**

**55kDa**

**180kDa**

**130kDa**

**70kDa**

**100kDa**

**55kDa**

**40kDa**

**35kDa**

**25kDa**

**55kDa**

**40kDa**

**35kDa**

**25kDa**

**70kDa**

**100kDa**

**180kDa**

**130kDa**

**Figure S7. The EMT and fibrosis of human IBECs were decreased after the p38 MAPK signaling pathway was blocked (full uncropped western blots).**


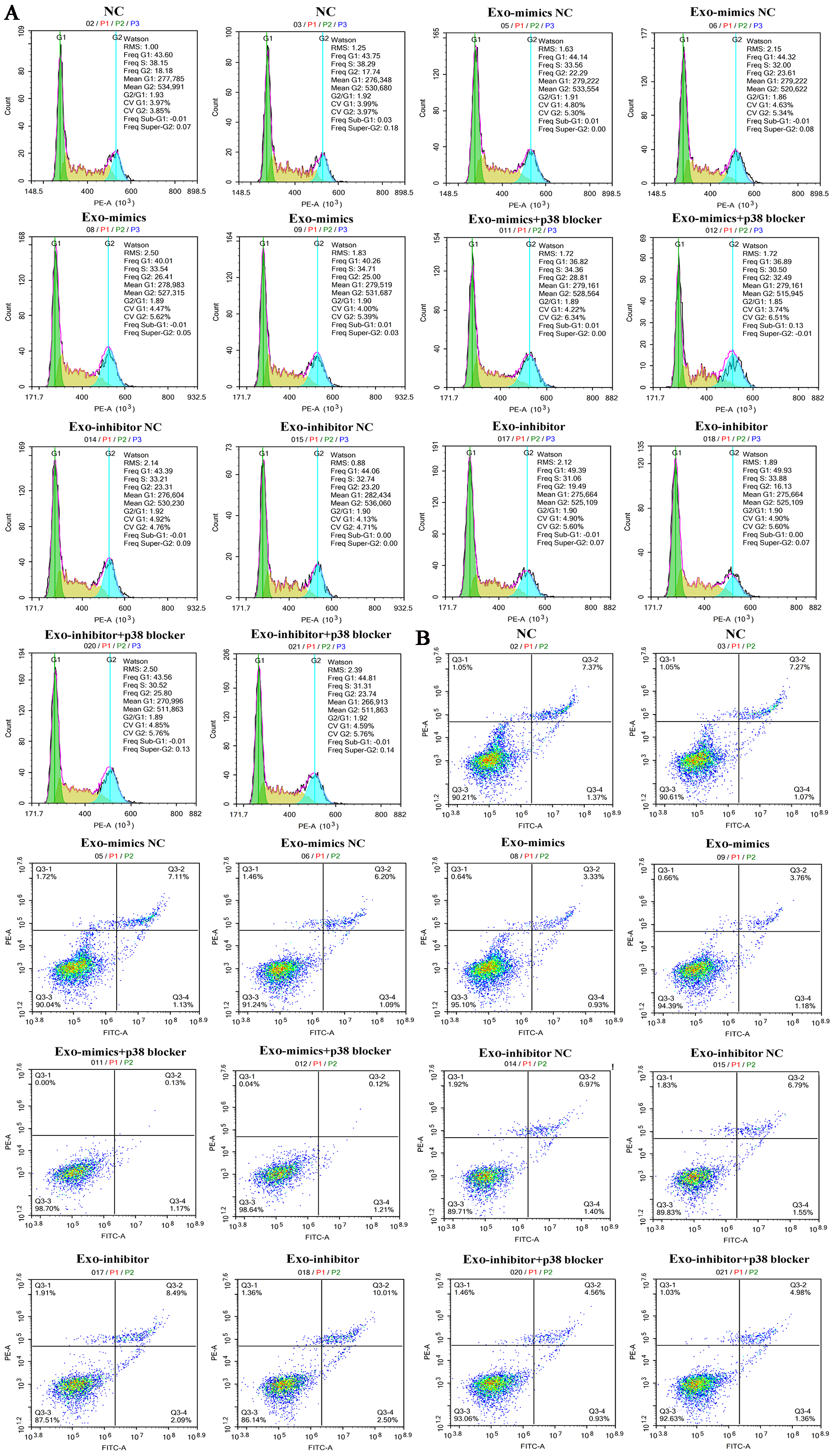


**Figure S8. Exosomal miR-122-5p affected the proliferation and apoptosis of human IBECs**

**via p38 MAPK signaling pathway. IBECs: intrahepatic biliary epithelial cells**


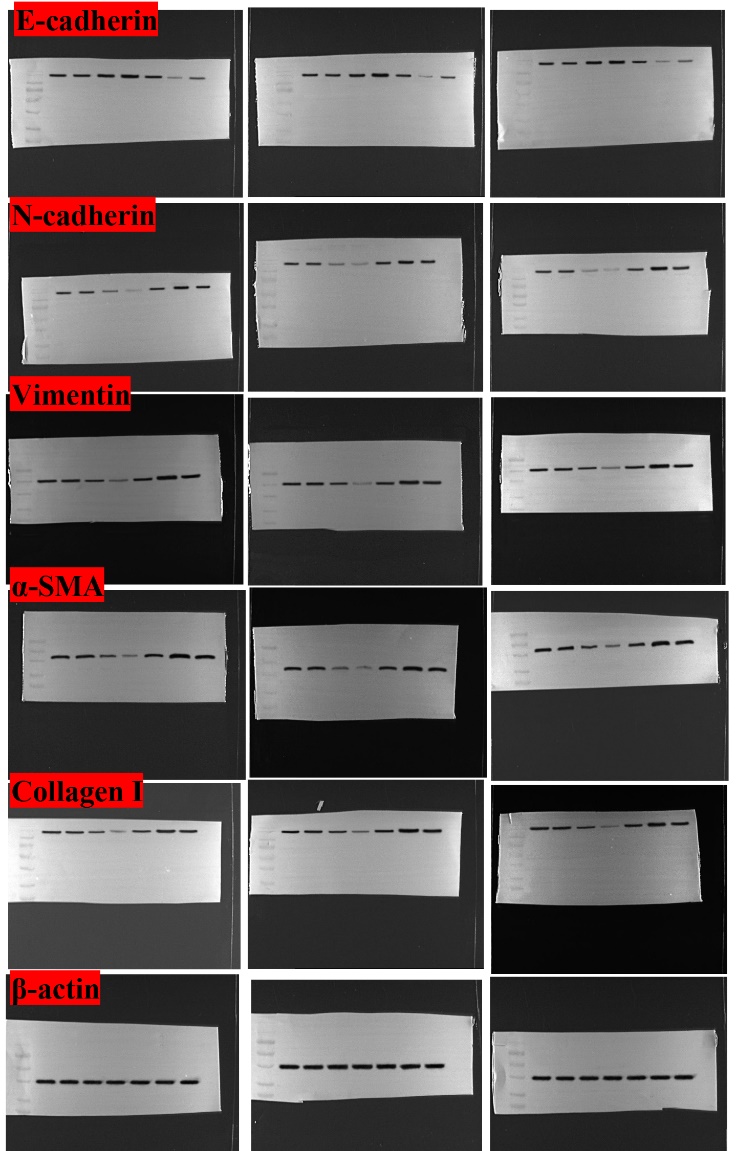


**25kDa**

**35kDa**

**55kDa**

**70kDa**

**40kDa**

**25kDa**

**35kDa**

**70kDa**

**40kDa**

**55kDa**

**100kDa**

**180kDa**

**130kDa**

**25kDa**

**35kDa**

**40kDa**

**55kDa**

**70kDa**

**25kDa**

**35kDa**

**40kDa**

**70kDa**

**55kDa**

**180kDa**

**55kDa**

**35kDa**

**100kDa**

**130kDa**

**70kDa**

**40kDa**

**25kDa**

**42kDa**

**130kDa**

**45kDa**

**55kDa**

**140kDa**

**25kDa**

**40kDa**

**135kDa**

**100kDa**

**180kDa**

**35kDa**

**55kDa**

**130kDa**

**70kDa**

**Figure S9. Exosomal miR-122-5p inhibits EMT and fibrosis of human IBECs via p38 MAPK pathway (full uncropped western blots).**


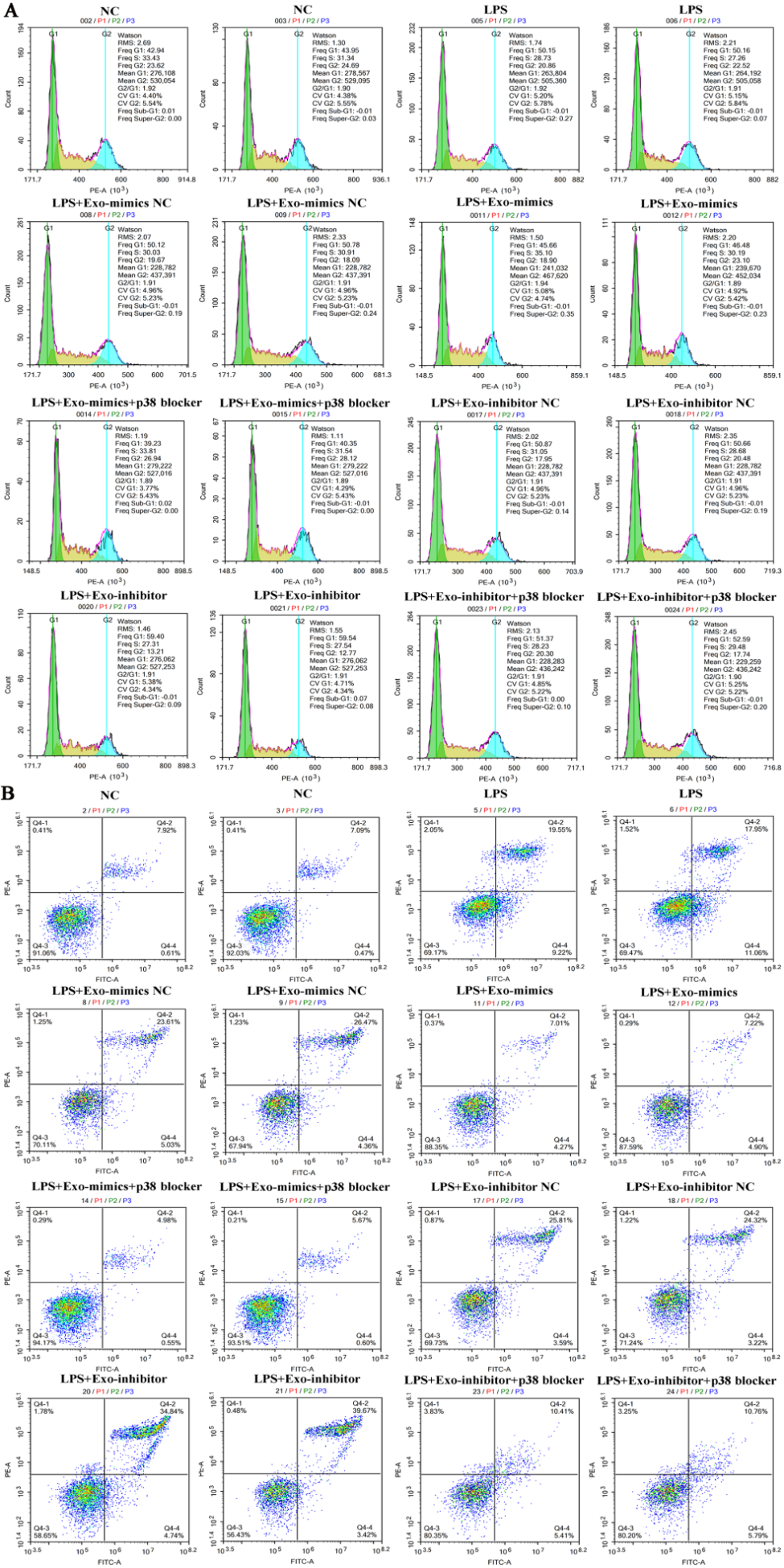


**Figure S10. Exosome miR-122-5p and p38 MAPK blocker synergistically promote proliferation and inhibit apoptosis in the human IBECs inflammatory model.**


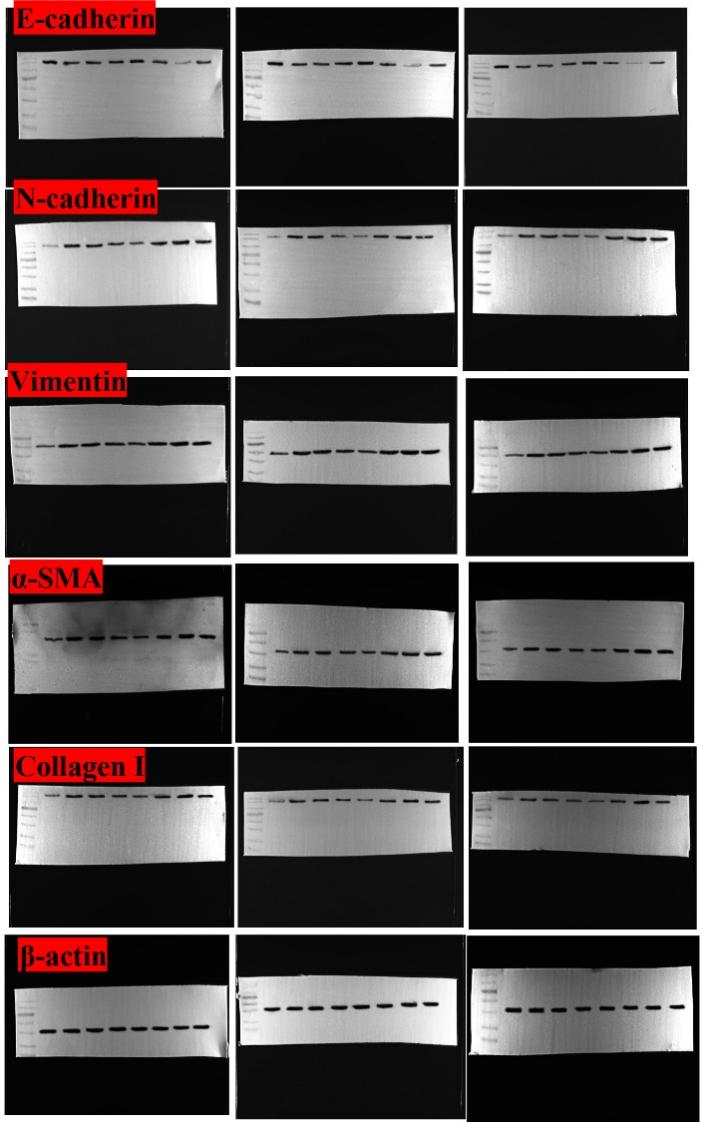


**70kDa**

**55kDa**

**25kDa**

**35kDa**

**40kDa**

**25kDa**

**70kDa**

**40kDa**

**35kDa**

**55kDa**

**100kDa**

**180kDa**

**130kDa**

**25kDa**

**35kDa**

**40kDa**

**55kDa**

**70kDa**

**25kDa**

**35kDa**

**40kDa**

**55kDa**

**70kDa**

**25kDa**

**35kDa**

**40kDa**

**55kDa**

**70kDa**

**130kDa**

**180kDa**

**100kDa**

**25kDa**

**35kDa**

**42kDa**

**130kDa**

**45kDa**

**140kDa**

**55kDa**

**135kDa**

**100kDa**

**180kDa**

**40kDa**

**55kDa**

**130kDa**

**70kDa**

**Figure S11. Exosomal miR-122-5p and p38 MAPK blocker synergistically inhibit EMT and fibrosis in the human IBECs inflammatory model (full uncropped western blots).**


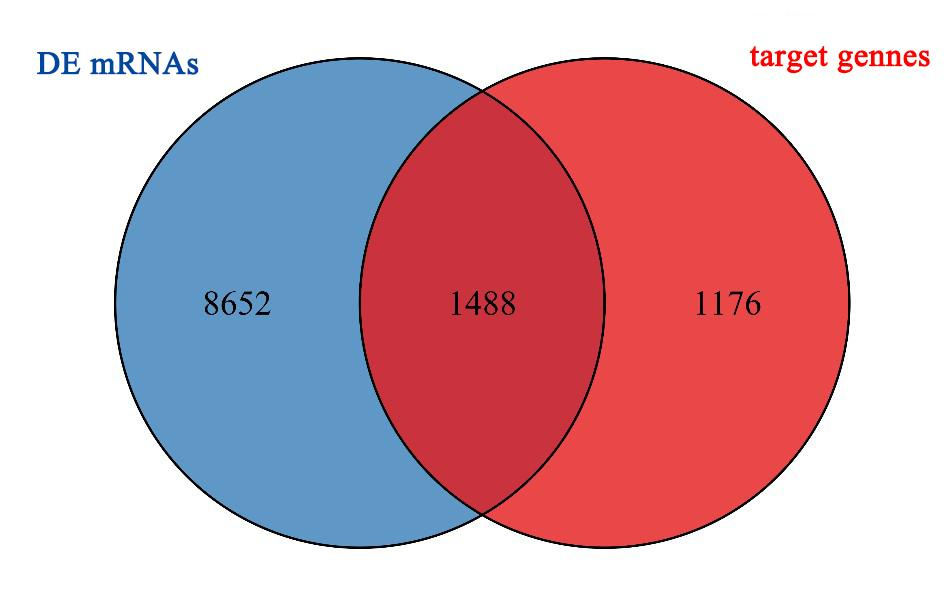


**Figure S12. Venn maps of overlapping genes between predicted genes and mRNA sequencing results.**


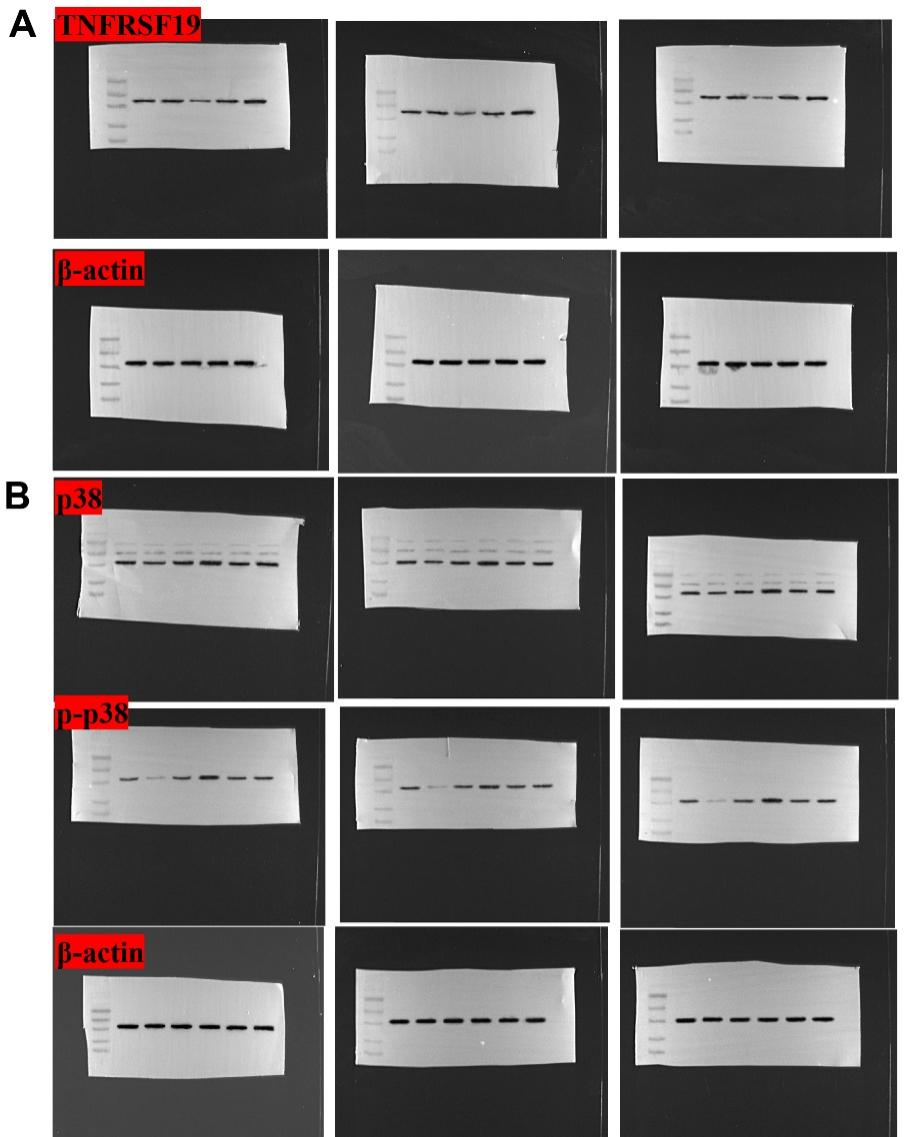


**70kDa**

**55kDa**

**35kDa**

**25kDa**

**40kDa**

**55kDa**

**25kDa**

**35kDa**

**40kDa**

**70kDa**

**35kDa**

**25kDa**

**70kDa**

**55kDa**

**40kDa**

**25kDa**

**35kDa**

**40kDa**

**55kDa**

**70kDa**

**42kDa**

**35kDa**

**25kDa**

**40kDa**

**55kDa**

**70kDa**

**42kDa**

**42kDa**

**43kDa**

**46kDa**

**Figure S13. A: MiR-122-5p negatively regulated the expression of TNFRSF19;**

**B: TNFRSF19 siRNA can reverse the increase of p38 and p-p38 protein caused**

**by miR-122-5p inhibitor (full uncropped western blots)**


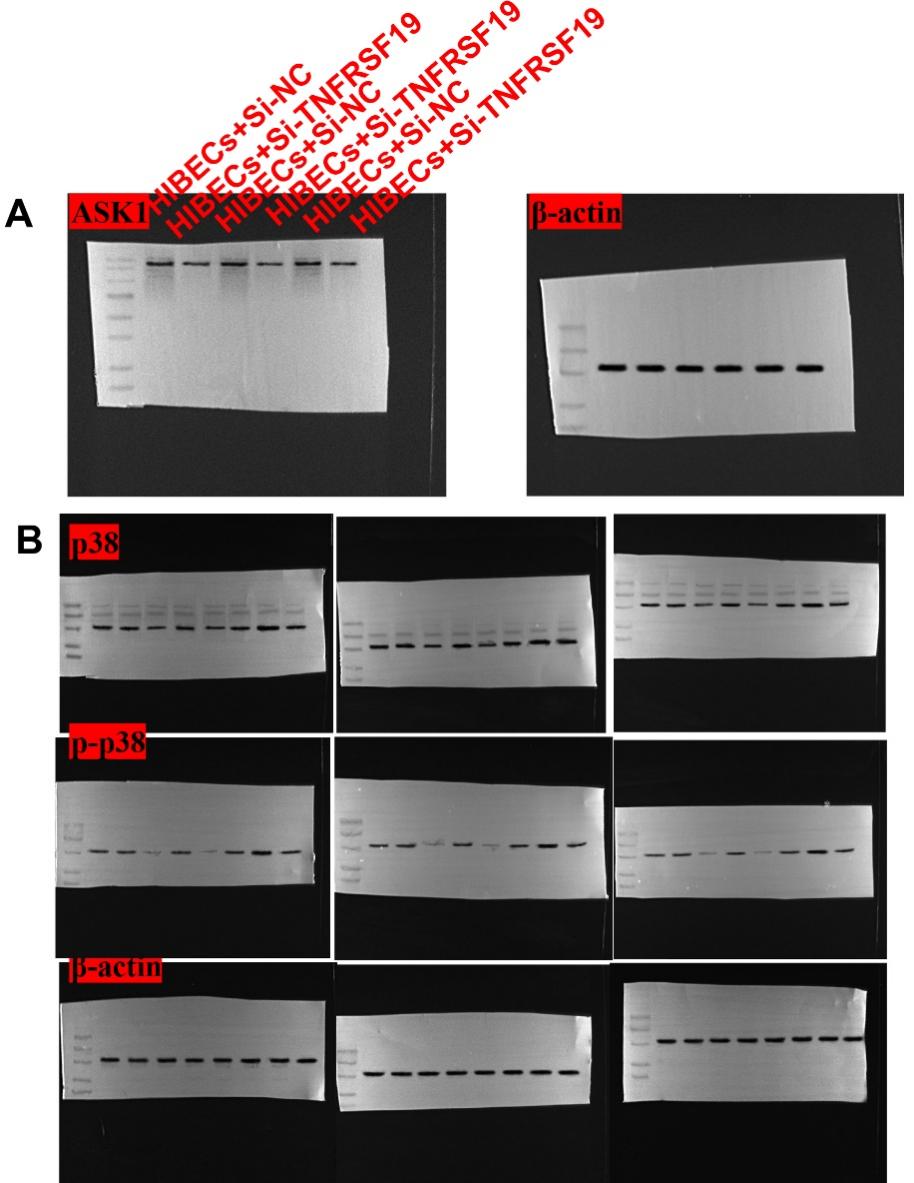


**25kDa**

**35kDa**

**40kDa**

**55kDa**

**70kDa**

**70kDa**

**55kDa**

**40kDa**

**35kDa**

**25kDa**

**70kDa**

**55kDa**

**35kDa**

**40kDa**

**25kDa**

**25kDa**

**40kDa**

**25kDa**

**35kDa**

**40kDa**

**55kDa**

**70kDa**

**100kDa**

**130kDa**

**180kDa**

**42kDa**

**43kDa**

**42kDa**

**155kDa**

**Figure S14. A: TNFRSF19 siRNA down-regulates ASK1 levels;**

**B: TNFRSF19 regulates p38 MAPK signaling pathway through ASK1 (full uncropped western blots).**


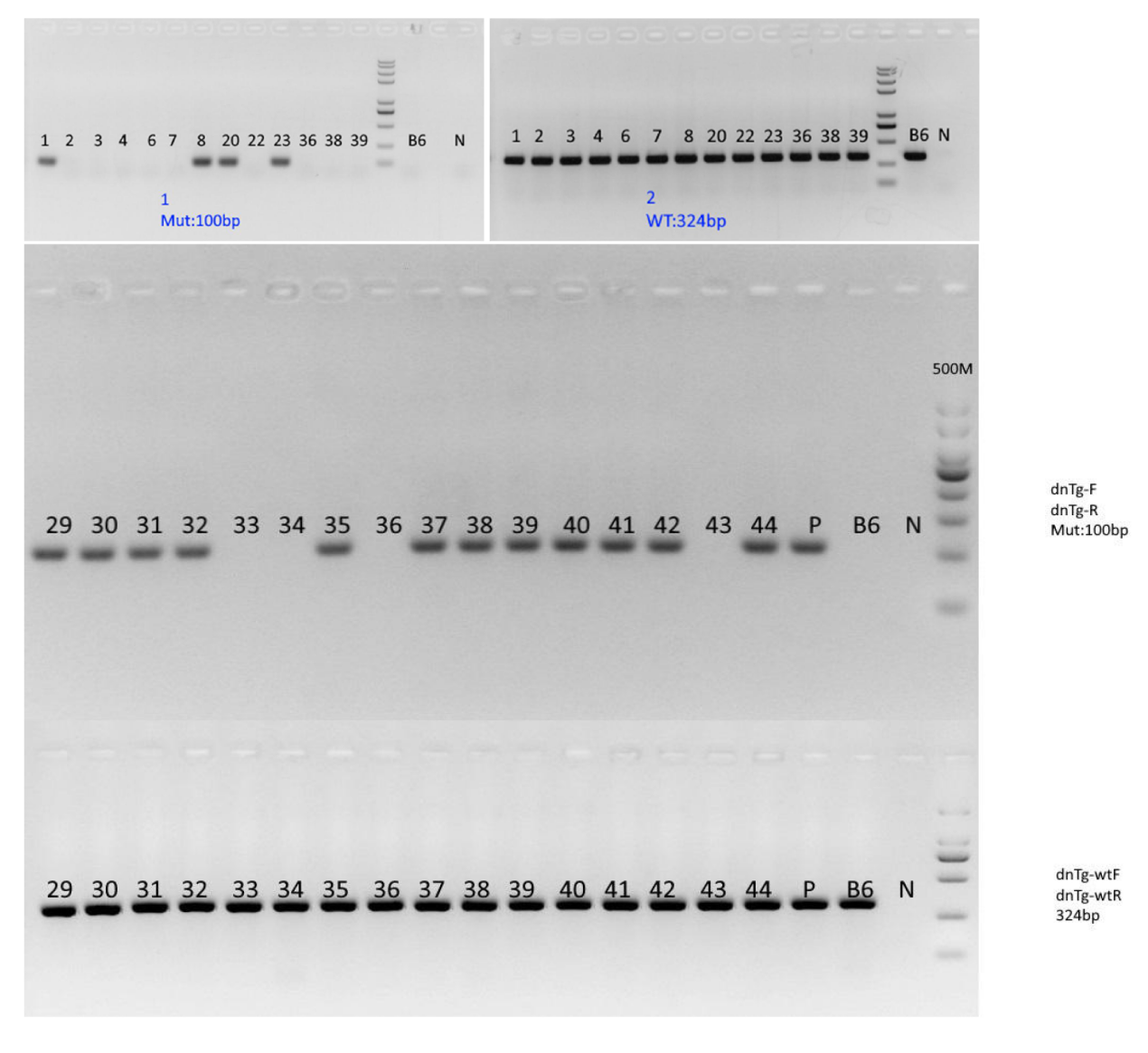


**Figure S15. Gene identification of PBC model mice**
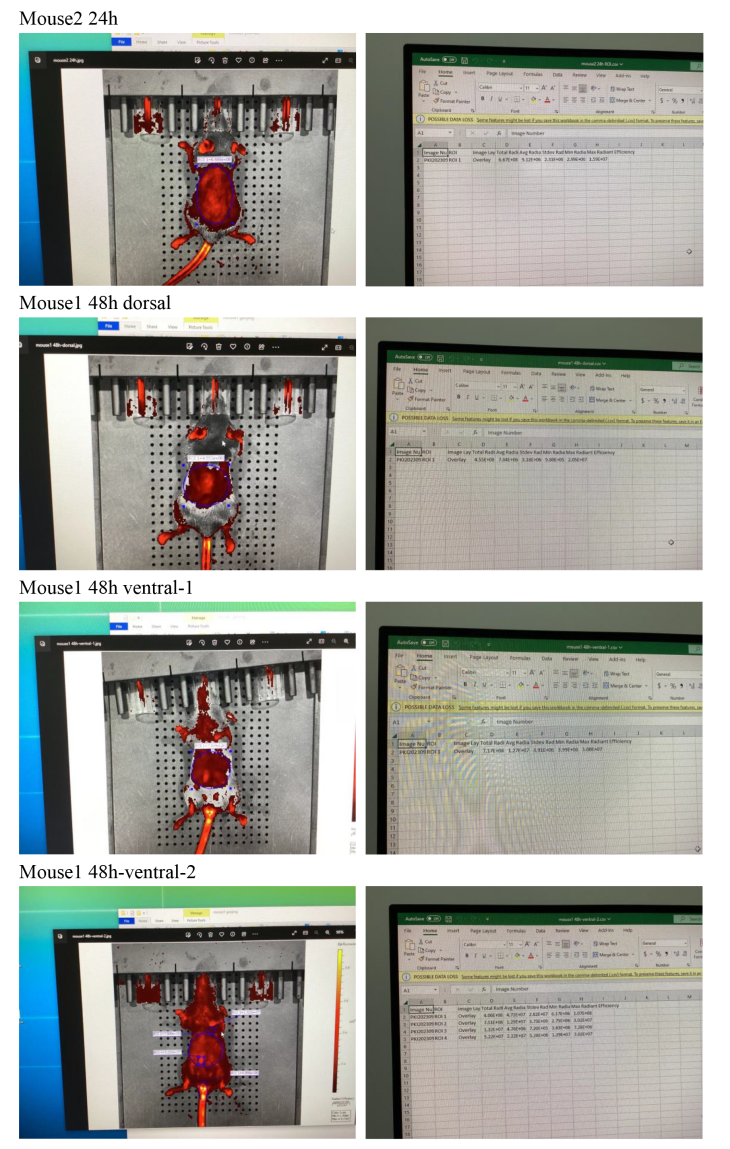


**Figure S16. Preliminary experiment of exosome injection in tail vein**

**Table S1. Primer sequences**

| **Genes** | **Size（bp）** | **Forward primer（5'→3'）** | **Reverse primer（5'→3'）** |
| --- | --- | --- | --- |
| Hu-U6 | 70 | CTCGCTTCGGCAGCACA | AACGCTTCACGAATTTGCGT |
| hsa-miR-122-5p | - | TGGAGTGTGACAATGGCGC | AGTGCAGGGTCCGAGGTATT |
| hsa-miR-122-5p RT | - | GTCGTATCCAGTGCAGGGTCCGAGGTATTCGCACTGGATACGACCAAACA | |
| Mimics-NC | - | UUCUCCGAACGUGUCACGUTT | ACGUGACACGUUCGGAGAATT |
| Inhibitor-NC | - | CAGUACUUUUGUGUAGUACAA | |
| Mmu-miR-122-5p mimics | - | UGGAGUGUGACAAUGGUGUUUG | AACACCAUUGUCACACUCCAUU |
| Mmu-miR-122-5p NC | - | UUCUCCGAACGUGUCACGUTT | ACGUGACACGUUCGGAGAATT |
| Hu-β-actin | 96 | CCCTGGAGAAGAGCTACGAG | GGAAGGAAGGCTGGAAGAGT |
| Hu-collagen I | 137 | CACCAATCACCTGCGTACAG | GCAGTTCTTGGTCTCGTCAC |
| Hu-vimentin | 152 | AGCTAACCAACGACAAAGCC | CGCATTGTCAACATCCTGTCT |
| Hu-E-cadherin | 114 | ACCATCCTCAGCCAAGATCC | CGTAGGGAAACTCTCTCGGT |
| Hu-N-cadherin | 99 | ACCAGGTTTGGAATGGGACA | CATTGAGGGCATTGGGATCG |
| Hu-TNFRSF19 | 94 | TGAAGCCTTTCAGCCAGTTG | GTATCATGAGTCGGCCTGGA |
| Hu-si-TNFRSF19 NC | - | UUCUCCGAACGUGUCACGUTT | ACGUGACACGUUCGGAGAATT |
| Si-1 TNFRSF19-785-sense/antisense | - | AGAGACAGUUUAUGGAGAAUU | UUCUCCAUAAACUGUCUCUUU |
| Si-2 TNFRSF19-788-sense/antisense | - | GACAGUUUAUGGAGAAGAAUU | UUCUUCUCCAUAAACUGUCUU |
| Si-3 TNFRSF19-872-sense/antisense | - | GACCUCAGCUCCACGAAUAUU | UAUUCGUGGAGCUGAGGUCUU |
| Hu-ASK1 | 175 | TCACGGACACTGAAAGCAGA | CAAAGGTGTAGTTCCCAGTGC |

**Table S2. Detailed information of antibodies**

| **Antibodies/indicators** | **Manufacturer** | **Catalog No** | **Antibody source** | **Dilution ratio** | **Separation glue concentration** |
| --- | --- | --- | --- | --- | --- |
| β-actin | Zs-BIO | 19AW0505 | Goat Anti-mouse | 1:1000 | - |
| Goat Anti-mouse IgG | Zs-BIO | 142637 | - | 1:10000 | - |
| Goat Anti-Rabbit IgG | Zs-BIO | 139931 | - | 1:10000 | - |
| Vimentin | Affinity | 26t9913 | Goat Anti-mouse | 1:1000 | 10% |
| α-SMA | Affinity | 22p5934 | Goat Anti-Rabbit | 1:1000 | 10% |
| Collagen I | Affinity | 26m3150 | Goat Anti-Rabbit | 1:1000 | 8% |
| E-cadherin | Affinity | 18q6272 | Goat Anti-mouse | 1:1000 | 8% |
| N-cadherin | Affinity | 12j8872 | Goat Anti-Rabbit | 1:1000 | 8% |
| ASK1 | Affinity | 29r3489 | Goat Anti-Rabbit | 1:500 | 10% |
| P38 | Affinity | 10y0837 | Rabbit | 1:500 | 10% |
| P-P38 | Abcam | GRI3715-64 | Mouse | 1:1000 | 10% |
| TNFRSF19 | Affinity | 82t9786 | Rabbit | 1:500 | 10% |
